# Efficient measurement of dynamic working memory

**DOI:** 10.3758/s13421-025-01724-x

**Published:** 2025-04-25

**Authors:** Garry Kong, Isabelle Frisken, Gwenisha J. Liaw, Robert Keys, David Alais

**Affiliations:** 1 Department of Integrated Arts and Sciences, Hiroshima University, Hiroshima, Japan; 2 Waseda Institute for Advanced Study, Waseda University, Tokyo, Japan; 3 School of Psychology, University of Sydney, Sydney, NSW Australia

**Keywords:** Working memory, Dynamic working memory, Attention, Crowding

## Abstract

Working memory is the ability to maintain a limited amount of information after it has been removed from perception. It is a key cognitive ability, thought to play a role in other cognitive functions, including perception, attention and action. Given its importance, its accurate and efficient measurement is a major goal in working memory research. Here we introduce a novel working memory tracking paradigm, inspired by continuous psychophysics and multiple object tracking. Participants viewed a sequence of stimuli moving along variable paths and were asked to reproduce the path by tracing it on a touchscreen. This reproduction was then compared to the original stimulus to determine error and thus memory performance. Across three experiments, we found that this new method is efficient, reliable and powerful, with only ten trials per condition required for stable performance estimates. We have also shown that the method is only minimally affected by perceptual or attentional confounds. Most importantly, since performance was measured across the trial, this method also allows for the investigation of how working memory changes across time. By averaging equivalent time points across trials, we identified influences from both primacy and recency effects, and quantified performance around particularly important points along the motion path. The working memory tracking paradigm is therefore especially useful when experimental time is limited, experimental conditions are extensive or when the time-course is a key interest. The method also opens up the study of working memory with dynamic stimuli.

**Supplementary information:**

The online version contains supplementary material available at 10.3758/s13421-025-01724-x.

## Introduction

Working memory is a critical component of human psychology, enabling many key cognitive abilities. Both learning and intelligence are said to arise from working memory (Conway et al., 2003; Cowan, 2014), while many cognitive disorders are associated with low working memory ability (e.g., Forbes et al., 2009; Schretlen et al., 2007). Working memory is not a monolithic process but comprised of relatively independent processes (Baddeley & Hitch, 1974). For example, visuospatial working memory describes our ability to hold and manipulate visual and spatial information for short periods of time, even in the absence of visual stimulus. It has been linked to our ability to deploy attention (Kong et al., 2020; Olivers et al., 2006), make saccadic eye movements (Kong et al., 2021b; Schut et al., 2017), and perhaps even plan and execute motor functions (Seidler et al., 2012).

**Fig. 1:**
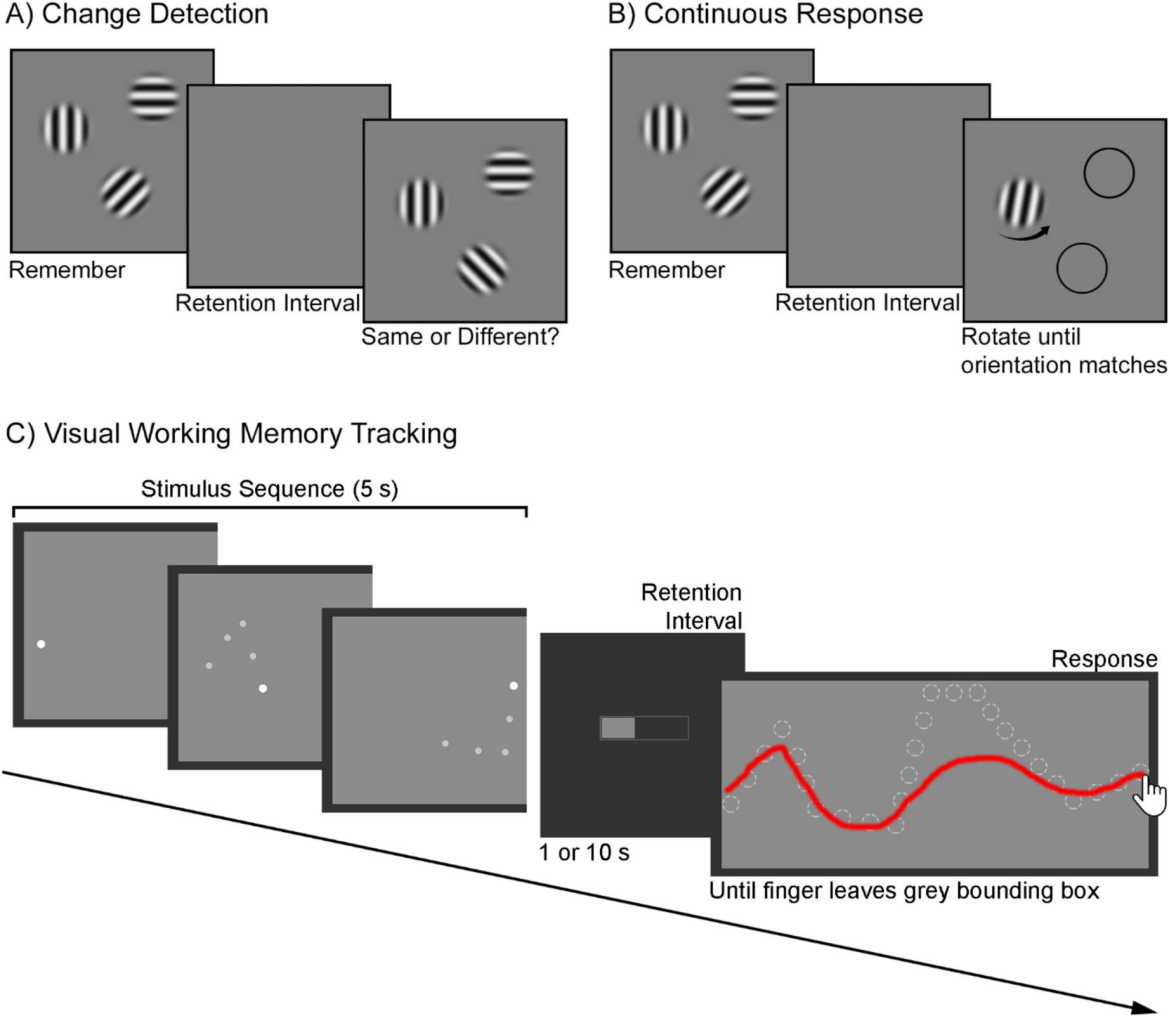
Visual working memory paradigms. Visual working memory paradigms. (**A**) Change detection. Participants remember the first frame, then compare the third frame their memory of the first. (**B**) Continuous report. Participants remember the first frame, then are cued to reproduce an element in that frame. (**C**) Working memory tracking. Participants remember a sequence involving a trail of dots zigzagging from one side of the rectangle to another, then reproduce the sequence. Dashed circles represent the actual path the stimulus took, while the red line represents the participants’ response in this example. In Experiment 1, the retention interval was 1 or 10 s

Given how vital an ability it is, measuring working memory accurately and reliably is a key endeavor in cognitive psychology research. Early working memory measurement was dominated by the memory span task (Jacobs, 1887), where participants are presented with a string of stimuli, usually numbers, before being asked to repeat them in a given order. The number that could be remembered was then used to calculate their working memory capacity (Miller, 1956). On the visual side, early research was via the change detection paradigm (Fig. 1A; Luck & Vogel, 1997; Rensink et al., 1997), where an array of visual stimuli is presented to the participant, before being removed. After a short retention interval, a probe array is then presented, and the participant asked whether the probe is the same as the first stimulus. Both these early methods have the advantage of being intuitive, with very little instruction required, and flexible, such that almost any stimulus can be presented without issue. However, this flexibility also reveals one of the problems with working memory measurement. Although we are supposedly measuring working memory capacity, there is always the chance that verbal, contextual and/or long-term memory influences are also being included in the working memory capacity calculation (Cowan, 2001). Chunking – pooling multiple items into a single memory item (Miller, 1956) – is perhaps the most famous example of such influences artefactually increasing working memory capacity measurements (Jones & Macken, 2015), but there are also examples of the reverse – unfamiliar and different-to-verbalize items seemingly taking up multiple items worth of working memory, artefactually reducing capacity measurements (Alvarez & Cavanagh, 2004). Indeed, the idea of measuring working memory capacity by number of items is likely itself an artefact of these tasks forcibly discretizing working memory, (Bays et al., 2009; Huang, 2010).

These early measures have since been updated to deal with some of these shortcomings, though never all at once. The complex span task involves interleaving a secondary task between the memory items of a digit span task, which supposedly prevents the type of processing required to allow for long-term memory influences (but see Mathy et al., 2018). Similarly, the n-back task (Kirchner, 1958) requires the participant to make a judgement about the stimulus that was *n* stimuli before the current one, such that the online nature of the task prevents contextual influences (Frost et al., 2021). Finally, the Corsi blocks task (Corsi, 1972) removes the possibility of verbal influences on working memory capacity by using physical blocks instead of digits as memory stimuli. The experimenter taps a number of blocks in a sequence, after which the participant reproduces the sequence. While an advance from the earlier tasks, the greater complexity of these tasks also opens up the problem of having to quantify the data collected into a working memory capacity score, with each task having multiple possible methods to express working memory capacity (e.g., Berch et al., 1998; Conway et al., 2005; Rouder et al., 2008).

On the visual side, the continuous report paradigm (Fig. 1B) is an evolution on the change detection paradigm, where instead of a same/different judgement, participants are asked to reproduce the probed item (Zhang & Luck, 2008). The difference between the presented stimulus and its reproduction is taken as a measure of the quality of visual working memory on that trial. The major advantage of this method is that the non-discrete response allows for statistical modelling of responses by keeping track of error, although at the cost of requiring the visual stimulus to be representable as a circular variable.

The many established paradigms have led to an explosion in working memory research. In recent times, however, their limitations have become apparent. Foremost amongst these is that we can only probabilistically assess the performance of a single trial. Even when guessing, a change detection trial has a 50% chance of being correct, and is therefore indistinguishable from a trial where memory quality was high. Similarly, a 20° error on a continuous report trial likely reflects a high quality of memory, but a guess still has a 5.6% chance of bettering it. This means, unfortunately, that it is impossible to determine whether performance is good or bad on any single trial. This in turn restricts us from conducting exploratory studies into what affects working memory performance. The typical way to conduct such a study would be to correlate trial performance with a second factor (or divide up trials into good vs. bad, and look for a difference). However, since the rank-order of trial performance is unstable (or the good group will inevitably have many bad trials mixed in and vice versa), the effect of the second factor needs to be exceedingly large to be detectable. Perhaps more importantly, the inability to quantify a single trial necessitates a large number of trials to compensate, leading to complex experiments with multiple experimental conditions either taking thousands of trials across multiple days (e.g., Bays et al., 2011), or three-digit numbers of participants (e.g., Bailey et al., 2008). The resources required also reduces the feasibility of comparing higher-order statistics, such as skew and kurtosis. Indeed, the few studies that have attempted this have tended to use only a single condition and compared the higher-order statistic to a null value (e.g., Fougnie et al., 2012; Yu & Geng, 2019).

These limitations of the established methods for measuring working memory have led to the recent development of alternatives. Of particular note is a betting game task (Jabar & Fougnie, 2022), which not only allows participants to make multiple attempts at a single trial, but also measures the shape of their belief distribution. The combination of these factors alleviates the problem of relying on a single point estimate, but at the cost of even lengthier experiments. Another innovative approach involves having participants view real world scenes, then simply asking them to draw what they remember (Bainbridge et al., 2019). This approach sidesteps the problem of point estimates completely, but at the cost of requiring a crowd of observers to score the responses, with all the problems of cost and subjectivity that such an endeavor entails.

In this study, we use a different approach by taking inspiration from continuous psychophysics (Bonnen et al., 2015) to introduce an evolution to the established methods, a working memory tracking paradigm. Continuous psychophysics is a paradigm in which a single, constantly changing stimulus is presented across a period of time, and the participant is asked to continuously make a judgement about it, in real time. The continuous response can then be compared to the original stimulus to produce a cross-correlogram, where the location of the peak of the cross-correlogram indicates the lag time between stimulus change and response, and the width indicates the accuracy. By collecting hundreds of data points across a trial sequence, the reliability of each trial is greatly increased, allowing a large reduction in the number of trials (and therefore, experiment time) required to get a stable estimate of performance. The continuous psychophysics paradigm, therefore, appears to be the perfect solution to the explosion of time required in working memory studies. However, there is a large problem with its implementation. By definition, a working memory task must be a response to a stimulus that is no longer being perceived, so the stimulus sequence must first be presented in full, then the response recorded afterwards. This in turn means that there is no guarantee that the stimulus and response have the same temporal characteristics, such as duration, pace and lag. This rules out the original cross-correlational analysis. Furthermore, a large part of working memory research deals with the increase of information load on capacity. As the continuous psychophysics paradigm was created for the presentation of a single stimulus, the paradigm will need to be adapted to allow for more.

Here we present three experiments in which we show how these problems can be overcome to adapt the continuous psychophysics paradigm for use in working memory studies. In so doing, we also show the benefits that the paradigm brings to working memory research. In Experiment 1, we show the paradigm’s ability to quantify memory recall performance on a trial-by-trial basis, by asking participants to memorize and recall the path of a single stimulus. In Experiment 2, we show that the resolution of recall performance is sufficient to easily distinguish between trials where the participant has some memory of the stimulus, and a lapse trial where the participant is purely guessing. Finally, in Experiment 3, we introduce a set-size manipulation and show that the task is indeed measuring working memory.

## Experiment 1

In this experiment, we adapt the continuous psychophysics paradigm to a working memory task with a single stimulus, show how working memory performance can be calculated, and showcase what additional information can be gleaned over and above the established working memory measurements. Participants view a single item that zig-zags from the left to the right of the screen and are then asked to reproduce the path of that item on a touchscreen monitor. Working memory quality can be inferred from how much the participant’s reproduced trace resembles the actual path of the item. As a basic check to ensure that the task is measuring working memory, the retention interval between the presentation of the stimulus and its reproduction was set randomly at either 1 or 10 s every trial. Finally, to ensure that variance in performance is due to working memory, and not the task itself, participants responded to the same six trials multiple times.

### Method

#### Participants

A total of 31 participants with an average age of 19.8 years, ranging from 18 to 22 years (six males) completed this experiment. We aimed for 30 participants as this was a new paradigm, and we wanted to err on the side of overpowering the experiment. An extra participant was included due to a procedural error. A Bayes Factor Design Analysis (Stefan et al., 2019) targeting an effect size of 0.85 (calculated from the *F*-value of the 4-s vs. 10-s duration comparison for the shape experiment in Zhang and Luck (2009), the most similar experiment to ours), found a minimum sample size of 23 was required for a 0.8 probability to find a Bayes factor over 3, with a default prior. All participants had self-reported normal or corrected-to-normal vision, had no tactile impairments and were naïve to the purposes of the experiment. Twenty-eight participants were right-handed. This research was approved by the University of Sydney Human Research Ethics Committee in accordance with the 1964 Helsinki Declaration. All participants gave written, informed consent before commencing the experiment and received course credit for their participation.

#### Apparatus and stimuli

Visual stimuli were created in MATLAB version 2020b using the Psychtoolbox (Kleiner et al., 2007). The stimuli were displayed on a 24-in. touchscreen monitor (Dell P2418HT, 60-Hz refresh rate, resolution of 1,920 x 1,080 pixels) from a viewing distance of approximately 57 cm. The background of the screen was a dark grey [RGB: 50 50 50]. The touchscreen input was sampled once every screen refresh.

The stimulus and trial sequence are shown in Fig. 1C. The target was a white [RGB: 255 255 255] circle with a diameter of 1° of visual angle, presented within a light grey [RGB: 140 140 140] 40.54° wide and 17.84° high rectangular bounding box. To aid in the temporal resolution of encoding, this stimulus was trailed by four smaller (0.8° diameter) dimmer [RGB: 200 200 200] circles, which denoted the position that the main stimulus had occupied at 0.25, 0.5, 0.75 and 1 s before the current point in time (see left panels of Fig. 1c). The stimulus travelled in a zig-zag path from the left to the right border of the light grey rectangle. Six unique paths were constructed randomly using the following restraints: the path would take 5 s; the Y coordinate of the start and end point was random; the stimulus would proceed through five randomly chosen turning points, at which it would change direction such that the path would create an angle that was greater than 20°, but less than 140°; the total distance the stimulus travelled would be between 54 and 67.5° of visual angle; and the minimum distance between turning points was 5.4° of visual angle.

#### Procedure

Each trial began with the screen showing the grey bounding box. After the participant indicated they were ready to begin the trial by pressing the spacebar, the target sequence was presented. When the target reached the right edge of the bounding box, the stimulus was removed, and replaced with a loading bar which indicated the time until the response was required. After 1 s or 10 s, the bounding box reappeared, and participants were asked to trace their finger over the path that the target took.

Participants performed 144 trials, made up of six repetitions of each of 12 paths (the six unique paths and a version where these paths were reflected about the horizontal axis), each with a retention interval of 1 s or 10 s. They also completed 12 practice trials, where the task requirements were explained to them. We also informed them it would be advantageous to fixate on the center of the bounding box, although we did not enforce this. Importantly, they were instructed to reproduce the path of stimulus as accurately as possible and at the same speed it was presented at. If reproduction of the path was shorter than 3.35 s or longer than 6.65 s, they were locked out for 3 s, told exactly how long their reproduction took, and to speed up or slow down accordingly. We observed the participant throughout the experiment and warned them verbally if we noticed they were using overt hand movements to aid their memory. Participants had the opportunity to take a break every 48 trials. Testing time for this experiment was approximately 45 min per participant.

#### Analysis

Responses were compared to the actual path of the presented stimulus. Given that the participants’ responses were not exactly 5 s long and could not be assumed to be at a constant velocity, we first had to match the response to the actual path to allow a direct comparison. To do this, we first divided the actual path into 30,000 equally spaced stimulus points. We then selected a single response sample to act as a starting point for the analysis. This sample was matched with the closest stimulus point on the path by Euclidean distance. The preceding response samples was then matched with the closest stimulus point that preceded the previously matched point. Similarly, the succeeding response sample was matched with the closest stimulus point that succeeded the previously matched stimulus point. In other words, the response sample at time *x* must be matched with a stimulus point that falls between the points that were matched to response samples at time *x*− 1 and *x*+ 1. This process was repeated until every response sample was matched to a stimulus point. Finally, this matching process was repeated for different response samples as a starting seed. The most appropriate match was defined as the one that produced the lowest spatial deviation score, calculated as lowest root mean squared error (RMSE) of the Euclidean distances between the matched response samples and stimulus points. This calculation was used (as opposed to mean absolute deviation) as it penalizes infrequent extreme values, for example, due to going in the wrong direction, more than it penalizes frequent smaller errors, for example, due to distortions to the spatial pattern in memory. We found considerable variation in RMSE based on the starting seed, and therefore ran most analyses using every possible data point as the starting seed. However, for computational reasons, for permutation analyses, the matching process used five equally spaced starting seeds. Supplementary Fig. 1 (see Online Supplementary Material (OSM)) depicts a more visual walkthrough of this spatial matching process.

This method provides the most charitable spatial match between the stimulus path and the participants’ response, allowing us to closely analyze spatial differences between the two. Although the matching process acts on each data point almost independently, the process is able to handle small global spatial distortions that would be expected to arise from a working memory reproduction (e.g., Haladjian et al., 2010). Supplementary Fig. 2 (OSM) illustrates how a contracted, expanded or rotated spatial response still produces decent matches to the stimulus path. Furthermore, since the process makes no assumptions about response pacing, it is also robust against temporal irregularities, such as participants speeding up or slowing down on turns (Viviani & Terzuolo, 1982). Supplementary Fig. 3 (OSM) illustrates why the process is relatively unaffected by the differences due to constant, ballistic and accelerating movement types.

The method also allows us to average multiple trials together for an even more reliable representation of performance. This can be done in two ways, by averaging across trials based either on each time or space. To average based on time, we linearly interpolated the string of Euclidean distances to 100 samples. In effect, this calculates the average error at each percentile of time of the response. To average based on space, the stimulus path was divided into areas defined by the start, end and turning points. Each section was then divided into ten equal bins. Data points that were matched with stimulus samples in a given bin were then averaged to calculate the error. In effect, this method calculates the average error at equivalent areas of the stimulus path (see Supplementary Fig. 4 (OSM) for an illustration of these averaging procedures). Note that in Experiment 2, trials identified as lapse trials were excluded for the purpose of this analysis, as the matching process inevitably put all data points in the first bin for these trials.

In all calculations involving the combination of data within a participant, RMSE was used as the metric for performance as it penalizes large errors. However, when averaging data across participants, a simple mean was used for two reasons. First, the magnitude of the errors varies based on the participant, such that a large error compared to other participants may be a small error within that participant. Second, our two methods of averaging across participants involve dividing the trials into smaller samples, where RMSE becomes less reliable.

In addition to the spatial precision this matching process affords, it also gives us a more accurate estimate of how far into their response the participant is at any point in time. For example, if the participant completes the first half of their response in 1 s (as opposed to the 2.5 s it should take), then slows down to complete the second half in 4 s, it would be wrong to assume that the middle of their response was 2.5 s after the start of the response (as the middle was after 1 s). This spatial match, therefore, also enables us to analyze the amount of temporal deviation in their response.

The temporal deviation analysis was performed as follows. The spatial matching process pairs each response sample with a stimulus point on the stimulus line. Both the response sample and stimulus point are then assigned a completion value based on how far into the response and stimulus paths it was (out of 100). These assigned values are then subtracted to obtain the temporal deviation. For example, if a sample 1 s into a 4-s response is matched with a point 2.5 s into the 5-s stimulus path, the completion value of the response is 25 and the completion value of the stimulus is 50, giving a temporal deviation of 25. This process is repeated for all response samples. To quantify performance of a single trial, we took the RMSE of these temporal deviations. To average performance across trials, we linearly interpolated the temporal deviations to 100 samples, then averaged equivalent samples across all the trials (Supplementary Fig. 4 (OSM)). Trials identified as lapse trials were excluded from this averaging analysis as they would inevitably skew the average towards time expansion. Notably, the spatial and temporal deviation measures are weakly correlated (0.384 in Experiment 1), indicating that while there is some overlap between the two measures, they measure substantially different abilities. That said, lapse responses will inevitably lead to large deviations in both spatial and temporal domains. The correlation in Experiment 2, where lapse trials are more common, rises to 0.803. Excluding lapse trials reduces this correlation to 0.408.

Bayesian statistics were used to quantify the strength of the evidence, calculated using the bayesFactor package on Matlab (Krekelberg, 2020). Bayesian *t*-tests used a Cauchy prior with a scale of 0.707 and Bayesian correlations used a stretched beta prior width of 1.0. Permutation analyses were conducted to give a sense of what performance would be like if participants were randomly guessing on all trials. This was done by shuffling the trials for each participant without replacement (in effect, removing the data labels) and redoing the analyses, then repeating this process 10,000 times, recording the results each time. The 2.5 th percentile of all the recorded results was taken as the lower bound of this permutation test.

### Results and discussion

**Fig. 2:**
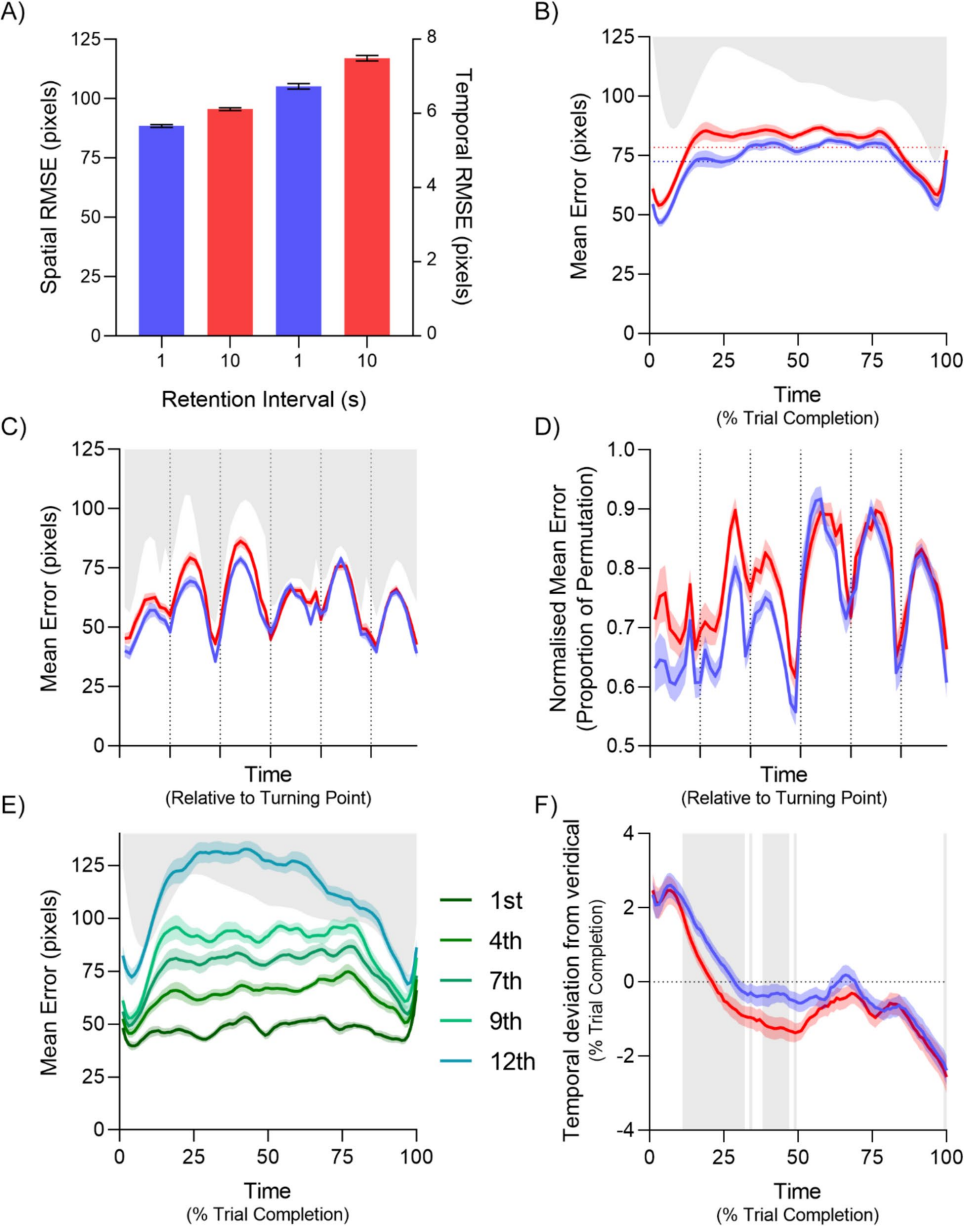
Results for Experiment 1. Blue represents 1-s trials, while red represents 10-s trials in all panels. (**A**) Mean spatial (left two bars) and temporal (right two bars) root mean square error (RMSE). (**B**) Mean spatial error across the trial. (**C**) Mean spatial error, rescaled to align turning points. (**D**) Mean spatial error when normalized by dividing by the mean permuted error, rescaled to align turning points. (**E**) Mean spatial error, ranked by performance. (**F**) Temporal deviation from veridical time across the trial. Positive values indicate time compression, while negative values indicate time expansion. All error bars and error bands represent within-subjects SEM (O’Brien & Cousineau, 2014). The grey region in Panel F depict points where BF_10_ > 3 for a difference between the two conditions. All other grey regions depict the 2.5 th percentile of permutated values, i.e., lower bound of the expected response when stimulus is unseen

Figure 2A depicts memory recall performance for the 1-s and 10-s retention intervals. A Bayesian *t*-test indicates that spatial performance was better in the 1-s trials than in the 10-s trials, BF_10_ = 1.58 x 10^7^. Similarly, temporal performance was also better in the 1-s trials than in the 10-s trials, BF_10_ = 6.23 x 10^5^. While it is no theoretical surprise that a longer retention interval leads to worse performance, even despite the lack of a set size, of special note is the effect size of d_z_ = 1.59 for spatial error, and d_z_ = 1.35 for temporal error.

The key benefit of this paradigm, however, is not simply its resolution, but that the resolution allows us to average across participants without also averaging over all of the data in a single trial sequence, enabling us to measure how working memory performance changes across time. Figure 2B depicts the average spatial error for the 1-s and 10-s conditions, averaged across the six unique stimulus paths. We found that performance was better after a 1-s retention interval predominantly in the first third of the trial, where the BFs of the difference at each time percentile ranges from 12 to 5.38 x 10^5^. Other periods of significant difference occur after this, but with weaker evidence.

Looking at general performance, we see that performance is best at the beginning and end of a response, consistent with a primacy and recency effect (e.g., Murre & Dros, 2015). Since our measure of time using this paradigm is continuous, we can also quantify the actual duration of these effects. If we define the effect as a difference from mean performance, then the primacy effect lasts until the 11 th percentile of trial completion in the 1-s retention interval, BFs ranging from 6.26 to 1.52 x 10^10^, and until the eight percentile in the 10-s retention interval, BFs ranging from 91.30 to 1.13 x 10^7^. Similarly, the recency effect begins at the 91 st percentile of trial completion for both retention intervals, BFs ranging from 3.86 to 1.62 x 10^8^, with the exception of the data point at the 100 th percentile, where performance reverts to the mean.

Another question we can ask is how the turning points of the stimulus path affect memory recall, as in Fig. 2C. Qualitatively, we see that spatial performance generally improves around a turning point, with local minima performance seen at the turning points. To quantify this effect, we compared performance at data points at and within two bins from a turning point (i.e., five bins, centered on the turning point), with data points not within this band. This confirmed the qualitative interpretation, with performance around turning points being lower, BF_10_ = 2.57 x 10^6^ for the 1 s retention interval and BF_10_ = 4.21 x 10^6^ for the 10-s retention interval. The performance benefit is especially clear once the variation in the difficulty across the trial is controlled for, as shown in Fig. 2D. This effect could suggest that participants are focusing more resources on remembering the turning points and reconstructing the path between; however, other strategies are conceivable.

One final spatial analysis this paradigm allows is the visualization of the natural variation in memory quality by ranking each participant’s response by its spatial RMSE across the whole trial, and averaging only across responses of the same rank. Figure 2E depicts the RMSE between the stimulus path and the first, fourth, seventh, ninth, and 12 th ranked responses averaged across the six unique paths and two retention intervals. We can see that there is a large amount of natural variability, which is expected, as the ranking was done post-hoc. Importantly, this suggests that variability in performance is due to working memory variability, not due to variation in the stimulus pathing. Such variability in memory recall has previously been observed, but has been difficult to visualize, as it typically manifested itself as a change in kurtosis of a Von Mises distribution of errors from the continuous report paradigm (Fougnie et al., 2012). Importantly, this variation indicates that memory quality is a continuous measurement, rather than a binary state as has been previously suggested (Zhang & Luck, 2009).

One additional temporal analysis can also be performed, investigating the average temporal error across the trial, as depicted in Fig. 2F. Generally, we can see that time is compressed in the first third of the trial, as the data is above the dashed 0 line on the left of the plot. Similarly, time is generally expanded in the last third of the trial. A compression in time means that participants are moving faster than the stimulus is progressing, while expansion means that participants are moving slower than the stimulus. Most importantly, performance in the 10-s trials qualitatively follows the same pattern as in the 1-s trials but is more exaggerated. Quantitively, the 10-s trials differ from the 1-s trials with a BF_10_ of 3 or greater between the 11 th and 32nd percentiles, and the 38 th and 47 th percentiles of trial completion.

It could be argued that this general trend is expected from the ballistic nature of human movements, i.e., we tend to accelerate at the beginning of, and decelerate into the end of, a planned movement. A more in-depth analysis of the Experiment 1 data shows that this is not the case. Since the stimulus paths in Experiment 1 were chosen from the same set of six, we can compute the temporal deviation patterns for each of the six paths and see if there is a pattern of compression/expansion around the time of the turning points (Supplementary Fig. 5 (OSM)). While each of the six paths generates a unique temporal deviation pattern, there appears to be no systemic relationship between the temporal deviation patterns and the turning points.

In summary, Experiment 1 has confirmed that this new tracking paradigm allows for the quantification and visualization of fine differences in the quality of working memory recall. In Experiment 2, we will show the flexibility of the task by generalizing it in two ways: increasing the set size, then changing the shape of the working memory task to accommodate this. These changes should in turn allow for even finer resolution of working memory differences.

## Experiment 2

Here, we repeat the previous experiment with the set size increased from one to three, presented simultaneously. This is important, as the field of working memory has historically focused on the number of items recalled (e.g., Luck & Vogel, 1997; Miller, 1956), so the ability to demonstrate set size differences will enable easier comparison between our paradigm and more traditional measures. Furthermore, a set size of one can be considered a special case, as the focus of attention is able to remain constantly on that one item, boosting performance for reasons not entirely related to working memory (Souza & Oberauer, 2016). Presenting multiple stimuli simultaneously, however, also introduces possible attentional and perceptual confounds, which will need to be overcome. Using knowledge from the multiple object tracking literature (Pylyshyn & Storm, 1988), we changed the stimulus space from a rectangle to a circle. This allowed memory stimuli to begin in the center of the circle and radiate outwards in different directions, to minimize potential stimulus overlap. Furthermore, the stimuli size increased as a function of the distance from the center, ameliorating the effect of perceptual crowding. Importantly, participants now viewed randomized stimulus paths, rather than repeating the same ones. The basic paradigm, however, remains unchanged – participants remembered the sequence of events, then reproduced it with a touchscreen.

### Method

#### Participants

A total of 12 participants with an average age of 22.9 years, ranging from 19 to 48 years (two males) completed this experiment. All were right-handed. Experiment 1 found that power was exceptionally high, so we reduced sample size to a more practical number. A Bayes Factor Design Analysis (Stefan et al., 2019) targeting an effect size of 1.2 found that a sample size of 12 is needed for to obtain a BF over 3 with a probability of 0.8.

### Apparatus and stimuli

**Fig. 3:**
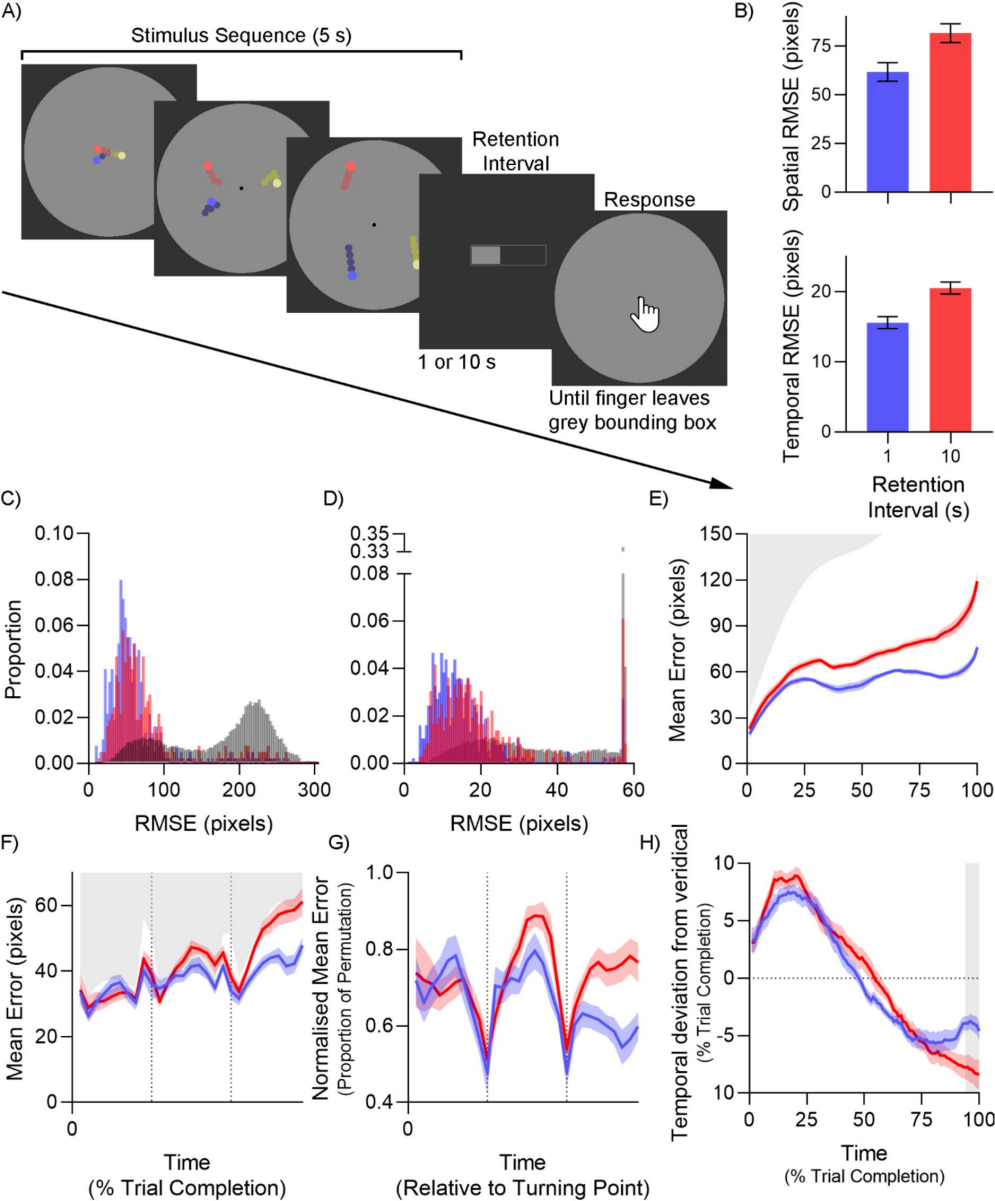
Method and results of Experiment 2. Note. Blue represents 1-s trials, while red represents 10-s trials in all panels. (**A**) Trial sequence for Experiment 2. (**B**) Mean spatial (upper) and temporal (lower) RMSE. (**C-D**) Histogram of spatial (**C**) and temporal (**D**) root mean squared error (RMSE) for 1-s retention interval trials (blue), 10-s retention interval trials (red) and permuted distribution (grey). (**E**) Mean spatial error across the trial. (**F-G**) Mean spatial error (**F**) and normalized spatial error (**G**), rescaled to align with turning points. (**H**) Temporal deviation from veridical time across the trial. Positive values indicate time compression, while negative values indicate time expansion. All error bars and error bands represent within-subjects SEM (O’Brien & Cousineau, 2014). The grey region in panel H depict points where BF_10_ > 3 for a difference between the two conditions. All other grey regions depict 2.5 th percentile of permutated values, i.e., lower bound of the expected response when stimulus is unseen

The stimulus was the same as in Experiment 1, with the following differences, as shown in Fig. 3A. Stimuli simultaneously radiated from the center of a grey circle with a radius of 9.40°. Each stimulus stayed within a different 120° wedge of the circle, such that the stimuli would not overlap after leaving the center and cause perceptual crowding. Furthermore, to negate the loss of visual acuity from the fovea to the periphery, the diameter of the stimulus increased as a linear function of the eccentricity from the center, from 0.8° at the center to 2° at the edge. To distinguish between the three stimuli, they were given different colors, chosen randomly from a pool of six colors: Red [255,100,100], Green [75,200,75], Blue [100,100,255], Magenta [200,50,200], Yellow [225,225,150] and Cyan [125,200,200]. The stimulus trails were dimmed versions of the same color. Given the reduced space for the stimuli to move, the length of a given stimulus’ path was between 8.1° and 18.92° and the minimum distance between turning points was removed. The number of turning points was also reduced to two, which were randomly chosen with no regard to previous location, i.e., the stimulus could double back. Each of the three stimulus paths per trial were created independently, such they would turn at different points in time except by coincidence. After stimulus presentation, a 0.8° diameter circle with the same color as one of the stimuli was presented at the center of the grey bounding circle. Participants were told to reproduce that path of the stimulus with that color.

#### Procedure

The trial sequence was the same as in Experiment 1. Participants performed 60 trials, made up of 30 trials for each retention interval (1 s or 10 s). They also completed 12 practice trials, where the task requirements were explained to them. Participants had the opportunity to take a break after 30 trials. Testing time for this experiment was approximately 20 min per participant.

### Results and discussion

As with the previous experiment, we first calculated the spatial and temporal error across every trial, averaged across participants for each condition, as shown in Fig. 3B. A Bayesian *t*-test found better performance in the 1-s condition in both conditions, BF_10_ = 4.69, d_z_ = 0.85 for spatial error, and BF_10_ = 23.46, d_z_ = 1.17 for temporal error. Observed power is lower this time, predominantly due to a single outlier, whose spatial performance was 2.9 SDs from the mean. Excluding this participant would have led to BF_10_ = 40.65, d_z_ = 1.38 for spatial error, and BF_10_ = 210.99, d_z_ = 1.79 for temporal error, which would have been more in line with the previous experiment.

The change to allow for 360° motion paths instead of a fixed direction increases the maximum error for each data point and allows for a new analysis, the identification of lapse rates. A histogram of all participants’ spatial error at the two retention intervals is shown in Fig. 3C. In the standard continuous report paradigm, guess rates have to be inferred from statistical modelling from an assumed distribution (Zhang & Luck, 2008), as the overlap between the information and no-information trials overlap completely. Here, informed trials are easy to separate from the lapse trials. Two distributions are readily apparent: a large one on the left representing informed trials, and a smaller one on the right representing lapse trials. To confirm this, we conducted a permutation analysis, denoted by the shaded grey histogram. The distribution on the right has increased, due to most trials now being uninformed. The distribution on the left is still present as the permutation analysis will invariably match the stimulus path with the correct response in some instances.

The same logic can be applied to analyze the temporal error, as shown in Fig. 3D. As with spatial error, two distributions are readily apparent, a large one on the left, and a small narrow spike at the end representing lapse trials. The permutation analysis again matches this interpretation, as the shaded grey bars show a short, wide distribution on the left, and a large spike on the right.

From this information, we can then model the distributions to determine whether the effect of a longer retention interval primarily reduces memory quality, resulting in a mean shift of the left distribution, or an increase in lapse trials, resulting in an increase in the right distribution. First, we modelled the permuted distribution using a two-normal distribution with five parameters: mean and SD of the left distribution, mean and SD of the right distribution and the relative weight of the two. This was done in order to extract the mean and SD of the right distribution (216.37 and 30.11, respectively, for spatial error, 57.30 and 0.11, respectively, for temporal error), which could then be passed onto the next analysis.

We then fit each participant’s RMSE distributions to the same model, with the following two changes: the mean and SD of the right distribution were fixed to the previously discovered values, and separate left-distribution mean and weight parameters were fit to the 1-s and 10-s retention interval data. This creates a seven-parameter model, with five free parameters and two fixed parameters. For spatial error, the model found a mean of the left distribution of 52.43 at 1-s and 60.75 at 10-s retention interval, and a Bayesian *t*-test found strong evidence of a difference here, BF_10_ = 28.10. Standard deviation of the left distribution was 20.49. Notably the lapse rate was low for all participants, with a mean of 6.39% for the 1-s and 14.66% for the 10-s retention interval, although the evidence for a difference was much weaker, BF_10_ = 1.69. For temporal error, the mean of the left distribution was 14.72 at the 1-s and 17.94 at the 10-s retention interval, with a Bayesian *t*-test finding strong evidence of a mean difference, BF_10_ = 28.40. Lapse rates were lower for the temporal error analysis, with a mean of 2.77% for the 1-s and 6.94% for the 10-s retention interval, again with weak evidence for a difference, BF_10_ = 1.67. Both analyses of the spatial and temporal error distributions suggest that the quality of working memory gradually reduces with time, rather than staying perfect until it disappears suddenly and completely, as advocated by Zhang and Luck (2009).

Looking at differences in spatial performance across time (Fig. 3E), we find that performance is better for the 1-s condition predominantly in the last third of the trial, BF_10_ ranging from 3.56 to 89.70. There is also a small portion of time between the 21 st and 32nd time percentiles where the difference is also evident, BF_10_ ranging from 3.29 to 19.03. Note the difference between Experiment 1, where the difference was mainly seen in the first third of the trial. This can likely be attributed to the different task demands. Since the stimuli now radiate from the center of a circle, the entry point of the stimulus is fixed in this experiment, whereas it varied on the y-axis in Experiment 1. Any effect of memory degradation will therefore be greatest towards the end of the trial. This manifests itself as a constantly increasing “problem space” (i.e., the number of possible responses), which we can see from the sharp increase in the grey shaded area representing the 2.5 percentile of the permutations in Fig. 3E. This task-induced increase in difficulty across the trial also makes it difficult to look for primacy or recency effects in this task.

We also investigated the effect of the turning points by realigning all the trials to their turning points. As with Experiment 1, we saw spatial performance generally improves around the turning point (Fig. 3F), despite every trial having a differently shaped stimulus path in this experiment. We quantified this by comparing the data points at and within two bins of the turning points with ones outside this band, and found evidence for the performance boost, BF_10_ = 3.27 for the 1 s and 28.82 for the 10-s retention intervals. The performance benefit is especially clear once the variation in the difficulty across the trial is controlled for, as shown in Fig. 3G.

Finally, we analyzed the amount of temporal error across time, as shown in Fig. 3H. The same overall trend as in Experiment 1 was observed here, with time compression in the first half of the trial, followed by time expansion in the latter half. However, strong evidence for a difference between the 1-s and 10-s retention intervals was only found in the last 6 percentiles of trial completion.

Combining Experiments 1 and 2, we can conclude that there is natural variation in the visual working memory quality (Fougnie et al., 2012). While there are many factors that influence this variation, retention interval is a large factor in the decay of this quality. Importantly, this new working memory tracking paradigm is able to visualize this natural variation because it enables working memory quality to be quantified with a single trial, instead of requiring the averaging across multiple trials. These conclusions obviously hinge on the assumption that this new paradigm is indeed measuring working memory. The last experiment, therefore, will test this assumption.

## Experiment 3

In this experiment, we will show that the working memory tracking paradigm used in the previous experiments are indeed measuring working memory. The key feature of working memory is that it is limited, such that memory performance will decrease as the memory load increases. Accordingly, in Experiment 3a we will show that RMSE increases as the set size increases. However, working memory is not the only cognitive ability that decreases with an increasing set size, most notably, perception and attention also exhibit this. Experiment 3b, therefore, will introduce a sequential version of the experiment that removes possible perceptual and attentional confounds. Finally, Experiment 3c will correlate performance on the two versions to determine the degree to which they measure the same cognitive resource. A strong correlation will show that despite the possible perception and attentional demands in the simultaneous version, the two versions are substantially measuring working memory performance. This will in turn enable future users of the paradigm to use the more practical simultaneous version without worrying about confounds.

### Method

#### Participants

A total of 20 participants with an average age of 20.6 years, ranging from 18 to 36 years (four males, two left handed) completed Experiment 3a, another 20 participants with an average age of 19.8 years, ranging from 18 to 26 years (five males, one left handed) completed Experiment 3b, and another 30 participants with an average age of 20.5 years, ranging from 18 to 39 years (six males, three left handed) completed Experiment 3c.

The sample size of Experiment 3a was almost doubled from Experiment 2 to allow for a more thorough sample size analysis later on (Table 2). Experiment 3b was increased to match.Importantly, Experiment 3c is a correlational study, making sample size especially important. Simulations showed that a sample size of 30 can find a correlation of 0.575 with a Bayes Factor greater than 3 at a probability of 0.8. As the aim is to show that the simultaneous and sequential presentation modes measure essentially the same thing, we judged a correlation this large as an appropriate target.

#### Apparatus and stimuli

**Fig. 4:**
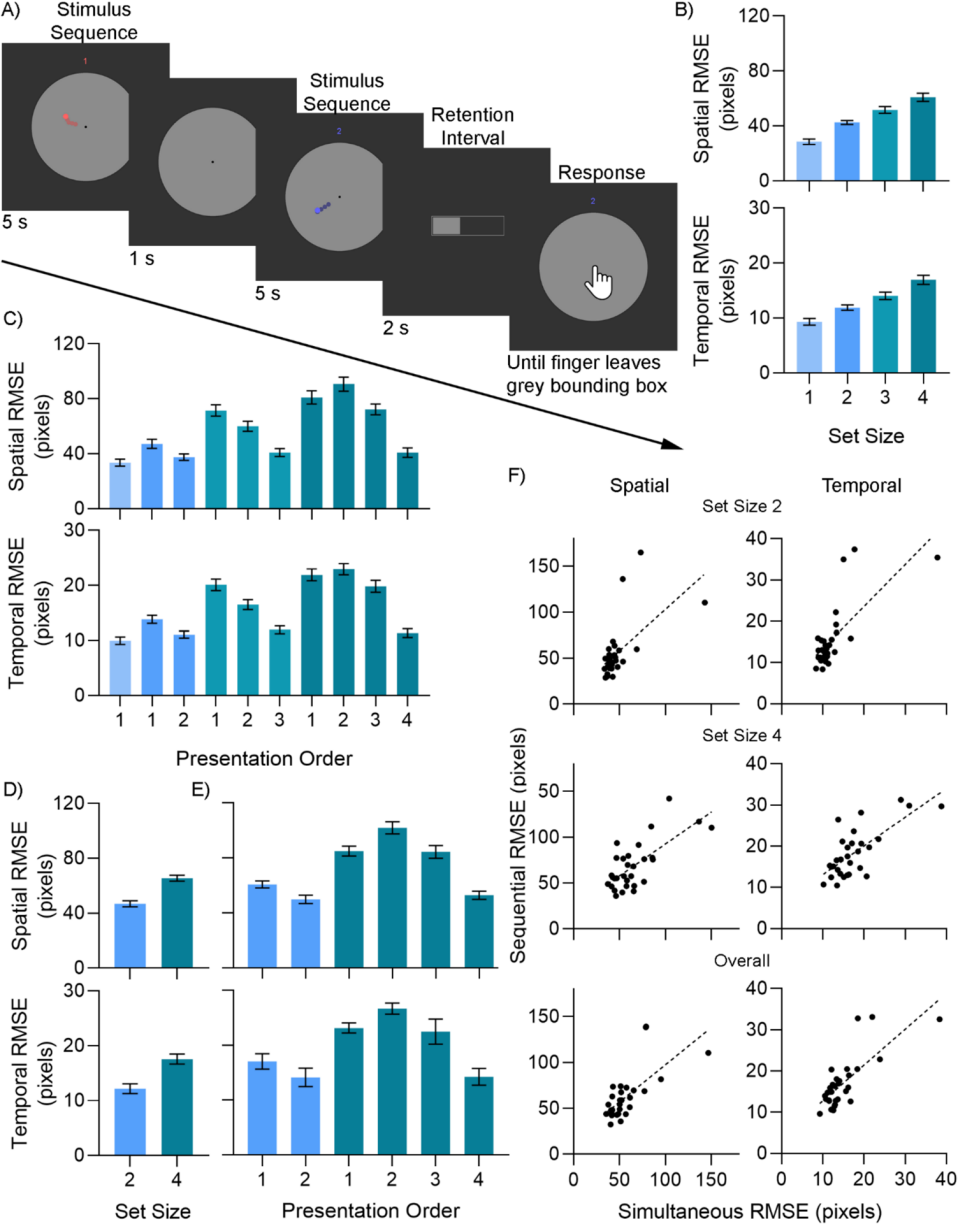
Method and results of Experiment 3. (**A**) Trial sequence for Experiment 3b. (**B**) Mean spatial (top) and temporal (bottom) root mean squared error (RMSE) for Experiment 3a. Colors used in this panel are consistent with subsequent panels, i.e., lighter blue is set size 1, darker teal is set size 4, etc. (**C**) Mean spatial (top) and temporal (bottom) RMSE for Experiment 3b. (**D**) Mean spatial (top) and temporal (bottom) RMSE for the simultaneous trials of Experiment 3 C. (**E**) Mean spatial (top) and temporal (bottom) RMSE for the sequential trials of Experiment 3c. All error bars are within-subjects SEM (O’Brien & Cousineau, 2014). (**F**) Scatterplots depicting the correlation between the simultaneous and sequential modes of presentation at set sizes 2, 4 and overall, respectively, and the line of best fit

Experiment 3a was the same as Experiment 2, with two exceptions. The number of stimuli presented varied from one to four, instead of being constant at three. To facilitate this change, the wedge of the circle that each stimulus could traverse was reduced to 90°. The retention interval was set at 1.5 s. Experiment 3b was a sequentially presented version of the experiment, as shown in Fig. 4A. Stimuli were presented in the same way as in the simultaneous presentation of Experiments 2 and 3a, with a few differences. Stimuli were presented one at a time, with a 1-s interval between stimulus presentations in which the grey bounding box is shown but with no stimulus. In addition to the color cue, a number was shown above the grey bounding circle during stimulus presentations, which indicated the current presentation order. At response, participants were cued with both the color of the stimulus and its number. Given the difficulty of the task, the retention interval was set to 2 s, as longer retention intervals facilitate memory consolidation (Luo et al., 2023), giving participants the opportunity to express their full memory of the stimulus. Experiment 3c involved both the simultaneous and sequential modes of presentation, with the same parameters as in Experiments 3a and 3b, with the exception that set size was limited to two and four. These two set sizes were chosen as we wanted to test the correlation on both a low and a high set size, but set size 1 is exactly the same task in the simultaneous and sequential presentation modes.

#### Procedure

The trial sequence was the same as in previous experiments. In Experiment 3a, participants performed 40 trials, made up of ten trials for each set size. In Experiment 3b, participants performed 100 trials, made up of ten trials for each combination of set size and ordinal position of the cued stimulus. In Experiment 3c, participants performed 120 trials, made up of 30 trials for each set size for the simultaneously presented conditions, and 60 trials made up for each combination of set size and ordinal position of the cued stimulus for the sequentially presented conditions. Participants performed all trials of one presentation type before moving onto the other, counter-balanced across participants. In all experiments, they completed 12 practice trials, where the task requirements were explained to them. Participants had the opportunity to take a break every 30 trials. Testing time per participant was approximately 10 min for Experiment 3 A, 45 min for Experiment 3B, and 45 min for Experiment 3 C.

### Results and discussion

#### Experiment 3a

Mean spatial and temporal RMSE for each set size is shown in Fig. 4B. All pairwise Bayesian *t*-tests strongly favored better performance on the lower set size condition, BF_10_ > 900, except weaker evidence for temporal error on the set size 1 versus 2 comparison, BF_10_ = 19.78, the 2 versus 3 comparison, BF_10_ = 8.67 for spatial error and BF_10_ = 2.09 for temporal error, and the 3 versus 4 comparison, BF_10_ = 1.94 for spatial error and BF_10_ = 3.05 for temporal error. Overall, this is good evidence that the working memory tracking paradigm with simultaneous presentation measures a limited-capacity ability.

Furthermore, the set size effect also allows us to address the concerns that participants might be using their motor system to rehearse the stimulus. Because of the motor requirements of the response, it is possible to perform well in this task by rehearsing the motor movements during stimulus presentation. However, such a strategy would only work at set size 1, meaning that the use of such a strategy should cause a disproportionate decrease in performance when comparing set sizes 1 and 2, when compared to other consecutive set size increases. Instead, we observe a relatively linear decrease in performance as a function of set size in both the spatial and temporal measures.

**Fig. 5:**
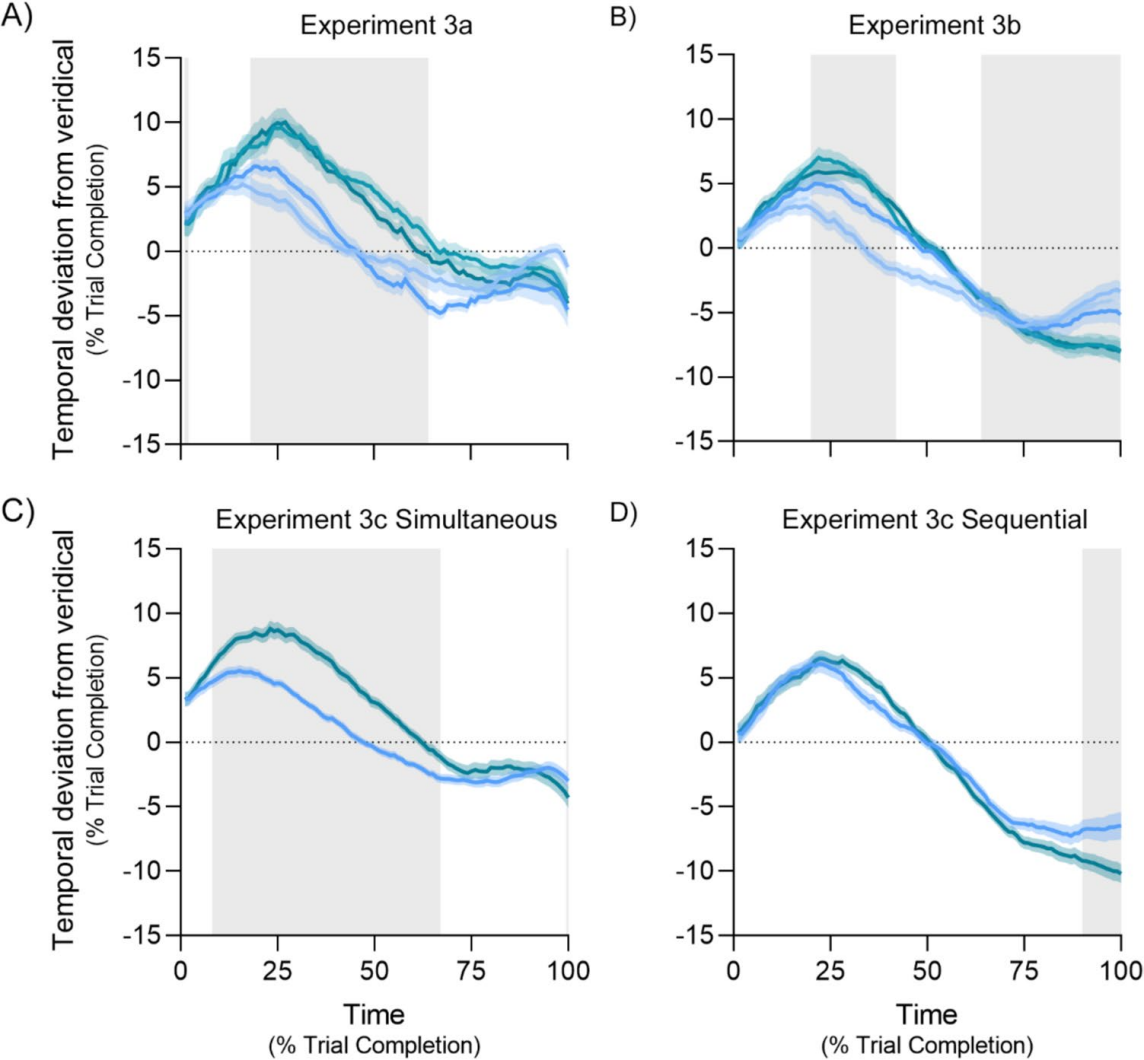
Temporal deviation from veridical time in Experiment 3. Pale blue, blue, teal and dark teal denote set sizes 1, 2, 3 and 4, respectively. Positive values indicate time compression, while negative values indicate time expansion. Error bands represent within-subjects SEM (O’Brien & Cousineau, 2014). Grey regions depict points where BF_10_ > 3 for a difference between set sizes 1 and 2 compared to 3 and 4 (panels **A** and **B**) or set size 2 compared to 4 (panels **C** and **D**)

Average temporal deviation across time is shown in Fig. 5A. As with the previous two experiments, the general trend of time compression in the beginning and time expansion at the end is evident. Qualitatively, it seems an increase in set size causes the magnitude of the deviation from veridical to become more extreme. For simplicity, the quantitative analysis was performed by averaging set sizes 1 and 2 and comparing that with the average of set sizes 3 and 4. We found evidence of a difference with BF_10_ > 3 for the first 2 percentiles, and between the 18 th and 64 th percentiles.

#### Experiment 3b

Mean RMSE for each condition is shown in Fig. 4C. Interpreting these results is more difficult than in Experiment 3a, as the presentation order clearly has an effect on performance. To judge the effect of set size on performance, we therefore chose to compare conditions where the presentation order is most comparable in recency. For example, the set size 1 condition would be compared to the most recently presented item in the set sizes 2, 3 and 4 conditions. Similarly, set size 2 conditions would be compared to the two most recently presented items in the set sizes 3 and 4 conditions. This was judged as the fairest way to compare across presentation order, as the previous experiments have shown that retention interval has an effect on memory recall.

For spatial error, Bayesian *t*-tests found weak evidence of better performance on set size 1 than set size 2, BF_10_ = 1.82, but stronger evidence of a difference in comparison to set size 3, BF_10_ = 8.85; and 4, BF_10_ = 16.90. Similarly, there was weak evidence that performance for set size 2 was better than for set size 3, BF_10_ = 2.63; and 4, BF_10_ = 252.38. Finally, performance on set size 3 was better than on set size 4, BF_10_ = 40.29. For temporal error, weak evidence was found against a difference in set sizes 1 and 2, BF_01_ = 1.18, stronger evidence for a difference in comparison to set size 3, BF_10_ = 6.37, and weak evidence of a difference in comparison to set size 4, BF_10_ = 1.56. For set size 2, weak evidence was found for a difference with set size 3, BF_10_ = 1.84, but strong evidence for a difference with set size 4, BF_10_ = 42.38. Finally, evidence was found for a difference in set sizes 3 and 4, BF_10_ = 4.38. In summary, strong evidence was found for better performance on lower set sizes when comparing non-consecutive set sizes, and weaker evidence found for consecutive ones. Taken as a whole, this is good evidence that working memory tracking with sequential presentation also measures a limited-capacity ability. Furthermore, since this task does not involve perceptual or attentional confounds, this ability is almost certainly working memory.

Average temporal deviation across time is shown in Fig. 5B. Again, we observe the general trend of time compression in the beginning and time expansion at the end. As with the simultaneous presentation, increasing set size qualitatively causes the magnitude of the deviation from veridical to become more extreme. The same quantitative analysis found evidence of a difference with BF_10_ > 3 between the 20 th and 42nd percentiles, and the last 11 percentiles.

#### Experiment 3c

Mean RMSE for each condition is shown in Figs. 4D–E. Performance on the set size 2 condition was better than for the set size 4 condition both when stimuli were presented simultaneously, BF_10_ = 1.30 x 10^4^ for spatial error and BF_10_ = 7.35 x 10^6^ for temporal error; and sequentially, BF_10_ = 31.09 for spatial error and BF_10_ = 7.08 for temporal error.

Average temporal deviation across time is shown in Fig. 5C and D. In both, we observe the general trend of time compression in the beginning and time expansion at the end. In the simultaneous presentation we see a clear effect of increasing set size to cause greater time compression during the trial, however, in the sequential presentation, we only see the same effect at the end of the trial. Quantitatively, we found evidence of a difference with BF_10_ > 3 between the 8 th and 67 th percentiles for the simultaneously presentation, and the last 11 percentiles for the sequential presentation.

More importantly, though, are the correlations between performance on the two tasks. Reliability estimates for the items used in the correlations are shown in Table 1. Scatterplots showing the relationship between the two presentation modes are shown in Fig. 4F. For spatial error, the correlation at set size 2 was *r* = 0.60, BF_10_ = 64.52; at set size 4 was *r* = 0.71, BF_10_ = 2.33 x 10^3^; and overall was *r* = 0.68, BF_10_ = 665.45. For temporal error, the correlation at set size 2 was *r* = 0.72, BF_10_ = 3.35 x 10^3^; at set size 4 was *r* = 0.73, BF_10_ = 3.70 x 10^3^; and overall was *r* = 0.77, BF_10_ = 2.24 x 10^4^. This suggests that the simultaneous and sequential tasks are essentially measuring the same cognitive ability, as about half the variance in one task is shared by the other. Interestingly, if the simultaneous presentation method contained perceptual and/or attentional confounds, we would expect these confounds to get worse as set size increased. Instead, we find that the correlation at set size 4 in fact trends higher than at set size 2, though we do not have the power to compare these statistically.

**Table 1:** Reliability estimates for items used for correlations in Experiment 3C

	Number of Items	Cronbach’s alpha	Spearman-Brown adjusted split-half correlation
*Spatial Error*			
Simultaneous Set Size 2	30	0.937	0.967
Simultaneous Set Size 4	30	0.892	0.958
Sequential Set Size 2	20	0.925	0.908
Sequential Set Size 4	20	0.820	0.776
Overall Set Size 2	50	0.952	0.949
Overall Set Size 4	50	0.921	0.944
*Temporal Error*			
Simultaneous Set Size 2	30	0.935	0.955
Simultaneous Set Size 4	30	0.890	0.943
Sequential Set Size 2	20	0.925	0.934
Sequential Set Size 4	20	0.808	0.598
Overall Set Size 2	50	0.957	0.832
Overall Set Size 4	50	0.921	0.864

*Note. n* = 30*.* When calculating Cronbach’s alpha, listwise exclusion was used where trials were missing due to input errors, leading to the loss of one participant for the sequential and overall conditions

Given that there is ample evidence that perceptual crowding and split-attention affect working memory encoding, why were these factors not confounds in the simultaneous presentation mode? We believe the seeming lack of effect in our experiments is due to the countermeasures we had in place to minimize the effect of such confounds, for example, confining each target to its own wedge to prevent overlaps and crossovers (Shim et al., 2008), and increasing the stimulus size with eccentricity to prevent crowding (Bouma, 1970). Indeed, these experiments cannot be taken as evidence that the simultaneous presentation of stimuli in working memory tracking will always be free of perceptual and attentional confounds, but only when such confounds are identified and managed.

#### Overall

Across the experiments in Experiment 3, we have shown that the working memory tracking paradigm exhibits a set size effect, which is a key characteristic of working memory. However, perceptual and attentional limits can cause performance to vary with set size, so we also showed that even when perceptual and attention limits are removed by having only one item at a time, the set size effect still exists. Finally, the set size effects on both the simultaneous and sequential presentation version of the task correlate strongly. Indeed, the observed correlation of 0.68 is near the theoretical maximum given the reliabilities of cognitive measurement, as even correlations between performance on the same change detection task only reaches a correlation of 0.76 (Xu et al., 2018). Of course, the large correlation is only evidence that the two tasks are measuring the same ability. However, given that this ability is systematically affected both spatially and temporally by set size (Experiment 3a and 3b) and retention interval (Experiments 1 and 2), it should be fair to say that this ability is working memory. Importantly, the correlation is evidence that any effect of any potential confounds on the simultaneous presentation version is limited, which is a large advantage from a practical point of view: 10 min to measure four set sizes is much shorter than 45 min.

## General discussion

This study introduces a new paradigm to measure working memory. This working memory tracking paradigm involves the presentation of a visual sequence that the participant is then asked to reproduce. In Experiment 1, we showed that performance on this reproduction varied greatly, but that this variation was not dependent upon the stimulus itself. In Experiment 2, we showed that this variance had a wide enough range that there was minimal overlap between trials where participants remembered something and trials where they were simply guessing. Importantly, the variance measured by the task is a reflection of working memory strength, as it covaried with set size (Experiment 3a and 3b), retention interval (Experiments 1 and 2), and remains even when the perception and attention load is removed by presenting the stimuli sequentially (Experiment 3b and 3c). This new paradigm offers some unique advantages over more traditional methods.

### Increased reliability

The greatest advantage to this new paradigm is the high degree of reliability. A task is reliable if equal performance on the task is quantified as the same value each time. We measure this using internal consistency measures, as in Table 1 from Experiment 3c. For comparison, complex span tasks report Cronbach’s alpha values ranging from 0.668 to 0.814, depending on the type and scoring method (Conway et al., 2005). Our range of 0.808 to 0.957, therefore, compares rather favorably.

The are several reasons why this new task ends up being so reliable, the most simply being that performance is quantified within a large range of measurement space: in Experiment 2 we see that performance ranges from 9 to 266. The size of the space is important as it allows for proper differentiation between trials where the participant remembers something, and when they just guess. Visual inspection of Fig. 3C shows that remembered trials tend to range from 0 to 120, whereas guesses typically range from 150 to 250. Since the worst performance never scores better than better performance, the task’s reliability is increased. This can be compared to span tasks or change detection, where performance is a binary state, either correct or incorrect. Complete guesses can still be judged as correct, and therefore be judged as on the same level as when the participant remembers the stimulus clearly. Of note though, is that continuous report tasks also have a large measurement space (− 180° to 180° on a color task), though there is some degree of overlap between good trials and guesses.

**Fig. 6:**
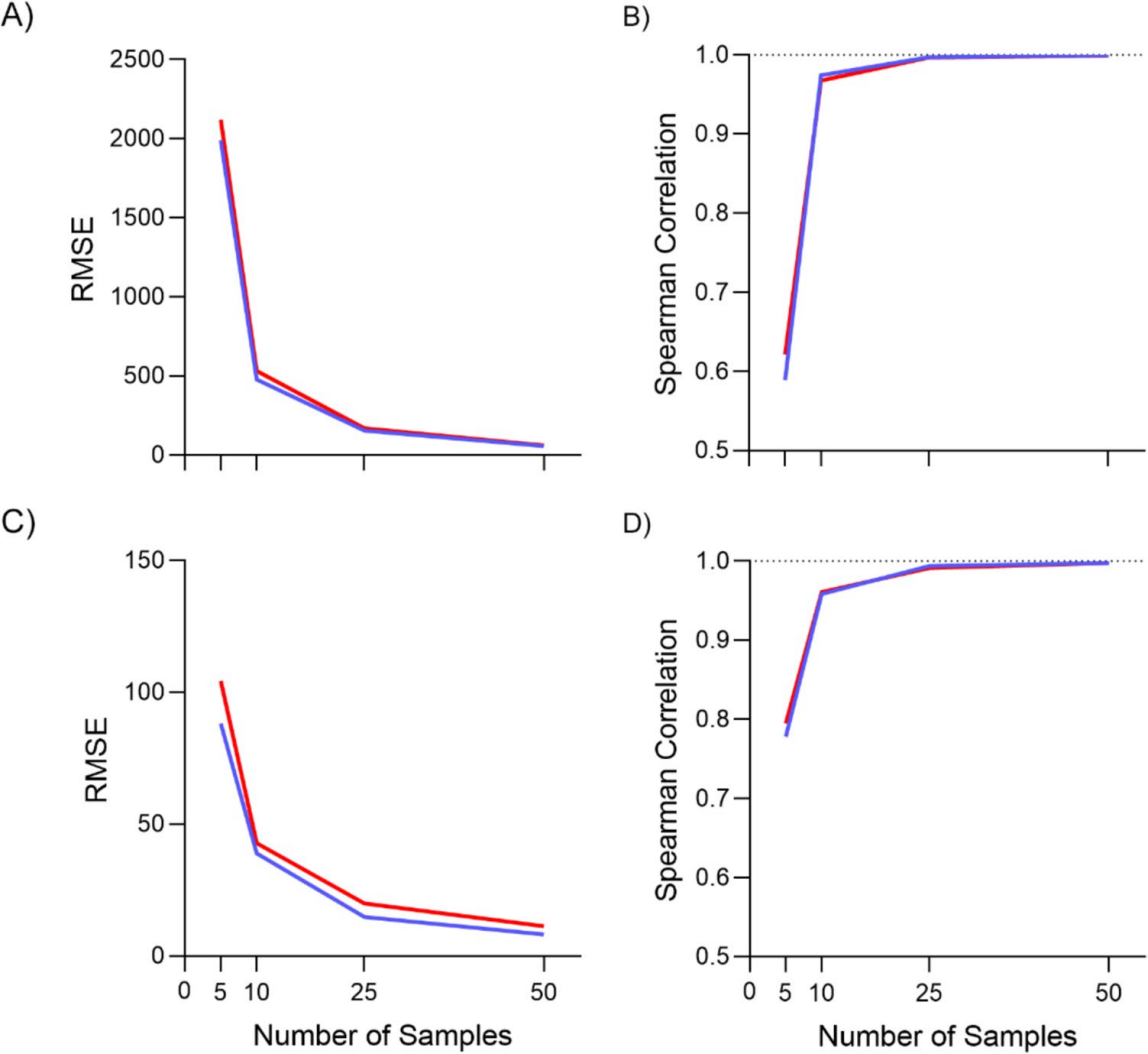
Effect of the number of data points. Blue and red lines represent trials with a 1- and 10-s retention interval, respectively. (**A** and **B**) Comparison between the spatial root mean squared error (RMSE) computed from a downsampled response and the full response. (**C** and **D**) Comparison of the temporal RMSE. The dotted line represents a Spearman correlation of 1, where rank order is perfectly preserved

The truly unique reason for working memory tracking’s reliability is that we are collecting hundreds of data points per trial. This continuous measurement allows performance to better reflect working memory state, as unintentional mistakes in responses, for example due to motor error, are corrected by the participant throughout the trial. Of course, although we collect hundreds of data points (60 data points per second), given the correlations between data points, especially those close in time, not every data point is of equal importance. To determine the relative significance of each data point, we reanalyzed the data from Experiment 1, using only 5, 10, 25 and 50 equally spaced data points from each trial and every participant. The results were assessed by comparing the RMSE between spatial and temporal RMSEs from the downsampled analysis and from the full set. For both spatial (Fig. 6A) and temporal (Fig. 6C) RMSE comparisons, we can see an exponential decay in the RMSE based on the number of data points, such that even at 50 data points, there is still a benefit to having the full set of data points. However, for most practical purposes, the exact accuracy score is less important than the relative accuracy, i.e., whether performance on one trial is better than another. To calculate this, we calculated the Spearman rank-order correlation, between the downsampled analyses and the full analysis. A Spearman correlation of 1 indicates that all trials were in the same order in both analyses. From Fig. 6B and D, we can see that for both spatial and temporal analyses, the rank order is mostly conserved with 25 data points. Therefore, despite the correlations between the data points, each trial is still gathering the equivalent of 25 data points in each five-second trial, the average of which will by definition increase reliability by reducing measurement error.

### Increased power

The most obvious advantage of increased reliability is that it increases the power of any experiment using this paradigm. In the current study, this manifests as large effect sizes, but future studies can take advantage of the power by reducing the sample size or number of trials each participant needs to complete. To illustrate what the increased power allows, we can run simulations to determine the sample size and number of trials required for the mean difference between two conditions to be above zero in 97.5% of runs, as shown in Table 2. To do this, we took the data of 5, 10, 15 and 20 random participants, with replacement, and calculated the mean RMSE difference between the two relevant conditions for each condition, and repeated this 10,000 times.

**Table 2:** Number of trials required for conditions to be significantly different as a function of sample size

Sample size	Experiment 2	Experiment 3a			
	Retention interval 1 s vs. 10 s	Set size 2 vs. 4	Set size 1 vs. 2	Set size 2 vs. 3	Set size 3 vs. 4
*Spatial*					
5	> 30*	8	3	> 30*	> 30*
10	7	4	2	18*	> 30*
15	4*	3	1	7	> 30*
20	3*	2	1	5	30*
*Temporal*					
5	24	6	> 30*	> 30*	> 30*
10	5	3	6	> 30*	> 30*
15	3*	2	4	> 30*	16*
20	2*	2	3	26	9

*Note.* * Denotes that either the sample size or number of trials required exceeds the actual sample size or number of trials used in the experiment, making the simulation unreliable

Given the small differences in working memory performance in consecutive set sizes, the most relevant calculations here are the comparisons in Experiments 2 and 3a between set sizes of 2 and 4. Here, we see that a sample size of 10, and less than ten trials per condition is sufficient to find a significant difference in either spatial or temporal error, which is less than 5 min of testing per participant. For comparison, Zhang and Luck (2009), used 12 participants and 150 trials per condition in their retention interval study. Similarly, Zhang and Luck (2008) used eight participants and 150 trials per condition, and Bays et al. (2009) used 12 participants and 50 trials per condition in their set size studies. While a single trial in this working memory tracking paradigm takes longer than in a change detection or continuous report paradigm (12 s vs. 1–2 s), the increased reliability makes up for it. This increased speed and experimental power makes the working memory tracking paradigm an obvious choice in settings where experimentation time is limited and in complex studies with many conditions. For example, when studying the effect of retention interval, a 10-s condition will only double the length of the experiment in this paradigm, whereas it would more than quintuple the length of a traditional experiment.

### Single trial quantification

The increased reliability of the task allows for something unique amongst working memory tasks: the ability to distinguish between trials where participants had a strong, weak or non-existent (i.e., lapse) working memory representation, as we did in Experiment 2. This is impossible for tasks where performance is binary, but also difficult for the continuous report task where performance is continuous. This is due to the fundamental problem of overlapping informative and lapse trials which make any quantification of memory probabilistic: a 10° error is probably reflective of clear memory, but there is a non-trivial chance that it was a guess, and therefore, reflective of no memory. This means comparisons between trials are accurate only when averaging across trials. In contrast to this, quantification of performance on the current paradigm is simple: a spatial RMSE of 20 shows that the participant has a clear memory of the stimulus, while 200 shows that the participant has no memory of the stimulus. We leveraged this ability to distinguish between trials where the stimulus was been weakly remembered and those where it is entirely forgotten in Experiment 2. We observed a rightwards shift in the distribution of informed responses as the retention interval increased from 1 to 10 s, indicating that memories gradually fade across time, rather than disappear suddenly, as had been previously proposed from evidence using continuous report paradigm (Zhang & Luck, 2009).

However, distinguishing between informed and lapse trials is only one way to leverage deterministic quantification. The distinction between strongly and weakly remembered trials is also of great importance. In this study, we have used this ability to visualize the inter-trial variability in Experiment 1, as shown in Fig. 2D. This variable precision in memory responses has been noted before in the literature (Fougnie et al., 2012) but has been difficult to visualize. These are, of course, only very basic ways to use the ability to quantify performance on a single trial. We believe that the true potential is that this allows for the correlation of a single trial performance with other factors, such as resting state signals, arousal markers, or other transitory states. This is especially important for neuroimaging studies, as trial-level correlations are much more practical than participant-level correlations.

### Investigation of temporal characteristics

The last major advantage of the ability to measure working memory quality in a single trial is that it opens up time as a new dimension to investigate in working memory. The study of the temporal dimension of working memory has previously been conducted either with a continuous sequence of a certain feature, followed by a response regarding the average or end-state of that feature (e.g., Chung et al., 2022; Zokaei et al., 2011) or by a presentation of stimuli across discrete steps in time, followed by a temporal order judgment (Corsi, 1972; Kong et al., 2021a). In both cases, the single response and discretized nature of time means that time is usually only included incidentally, and little can be concluded about the temporal aspects of working memory itself.

In contrast, the working memory tracking paradigm produces a continuous measure of memory recall across the course of a trial, which we used in Experiment 1 not only to observe primacy and recency effects, but also to quantify their duration. Furthermore, we observed that differences in performance due to retention interval manifest at different points in time, depending on the task demands. In Experiment 1, the effect of retention time was most pronounced in the first third of the trial, whereas in Experiment 2, it was the last third. More work needs to be done to tease out the exact reasons for such differences, but the fact that such differences even exist is proof that there is something to be learned from investigations into the temporal dimension.

Perhaps more important than the raw performance at certain temporal intervals is the variation of performance across time, and more specially, what causes this. In both Experiment 1 and Experiment 2, we noticed that performance improved around turning points in the motion path, reminiscent of the performance benefit when visual working performance is measured around a spatial landmark (Aagten-Murphy & Bays, 2019). Given that there is a spatial component to this task, it would not have been surprising to see a landmark effect in this experiment. However, spatial landmark effects have been found to disappear when the spatial landmark is present at encoding, but not at test. Again, more work needs to be done to identify the source of this performance benefit at the turning points, but it is good to see that this paradigm can identify these effects in the first place.

Finally, the continuous nature of the task also enables us to measure the temporal deviation of the participants’ response from veridical time. In all three experiments, we found a general tendency for the participants’ responses to be compressed in time at the beginning and expanded at the end of the trial. This general trend of time compression in the beginning is in line with anecdotal accounts in debriefing of participants wanting to record their response before they forgot it. Similarly, the time expansion at the end is in line with our generally observed trend of increased uncertainty at the end of the trial, as seen in Figs. 2B and 3E. However, both of these are mere hypotheses at this stage, as this question would be better answered by a study tailored to address it. For example, temporal deviation in general would be more easily studied in stimuli that varied more in speed and acceleration. The most important thing for the purpose of the current study is that these temporal deviations are meaningful, and this method enables its measurement.

### Content validity

Although not an advantage over the traditional methods, the working memory tracking paradigm was created with content validity in mind. It involves a wide range of abilities often attributed to working memory, allowing it to capture a large portion of the associated variance. For example, while the stimulus itself is spatial in nature (though note that any dynamic stimulus could theoretically be tracked), this spatial information needs to be integrated over time into a two-dimensional (2D) representation. This representation has a shape, orientation and, in Experiments 2 and 3, a color, that all need to be integrated into an object file (e.g., Hollingworth & Rasmussen, 2010). However, a 2D representation is not sufficient for proper maintenance, as the temporal portion of the task necessitates rehearsal (but see Oberauer, 2019). Finally, the length of time required during the recall process forces the participant to constantly compare their current response with their memory, so that they can self-correct their mistakes.

Also of note, the working memory tracking task inherently excludes the effects of verbal and long-term memory. Like the Corsi blocks task (Corsi, 1972), the stimuli are purely spatial in nature, removing verbal influences without resorting to articulatory suppression (Murray, 1967). Similarly, the randomized and, in Experiments 2 and 3, unrepeated nature of the stimuli eliminates the benefit of chunking without relying on a secondary task, as in complex span tasks (Conway et al., 2005). Controlling for such alternate strategies has been a major consideration in the study of the developmental time course of working memory (Cowan, 2016). The simplicity of the paradigm may prove to beneficial for such studies, though consideration will need to be made for the motor aspect of the task.

### External validity

Working memory is the storage of information that was recently present in the world but is no longer. Such a definition presupposes that there is a dynamism to the visual stimuli in the world, constant change that the working memory system must capture. Despite this, most of the established methods of measuring working memory are static, using unchanging stimuli for encoding, and taking only single measurements at test. While we have learnt much from these established methods, the dynamism of the working memory tracking paradigm, at the very least, improves the external validity of measures. It is quite possible that future experiments will find that our current knowledge of the field is unchanged with introduction of dynamic stimuli. However, it is just as likely that we have only been studying an edge case of working memory in a static world. In this case, this new paradigm unlocks our ability to surpass this edge case and investigate working memory in a dynamic world.

## Supplementary information

Below is the link to the electronic supplementary material.

Supplementary file1 (DOCX 990 KB)

## Data Availability

Data used for the analyses can be accessed at: https://osf.io/wgz2b/?view_only=983ae76566db4d7c8bfc19bee9dbaf08

## Code availability

The above repository also contains Matlab code to run a generic visuospatial working memory tracking experiment, as well as code to analyse the data.
