# Supplementary material for: Efficient measurement of dynamic working memory
Source: Mem Cognit. 2025 Apr 25;53(8):2535–55. doi: 10.3758/s13421-025-01724-x (PMC12695947; doi:10.3758/s13421-025-01724-x)
Supplement: Supplementary file 1 — Supplementary file1 (DOCX 990 KB) [file 13421_2025_1724_MOESM1_ESM.docx]

**Supplementary Materials**

**Supplementary Figure 1**


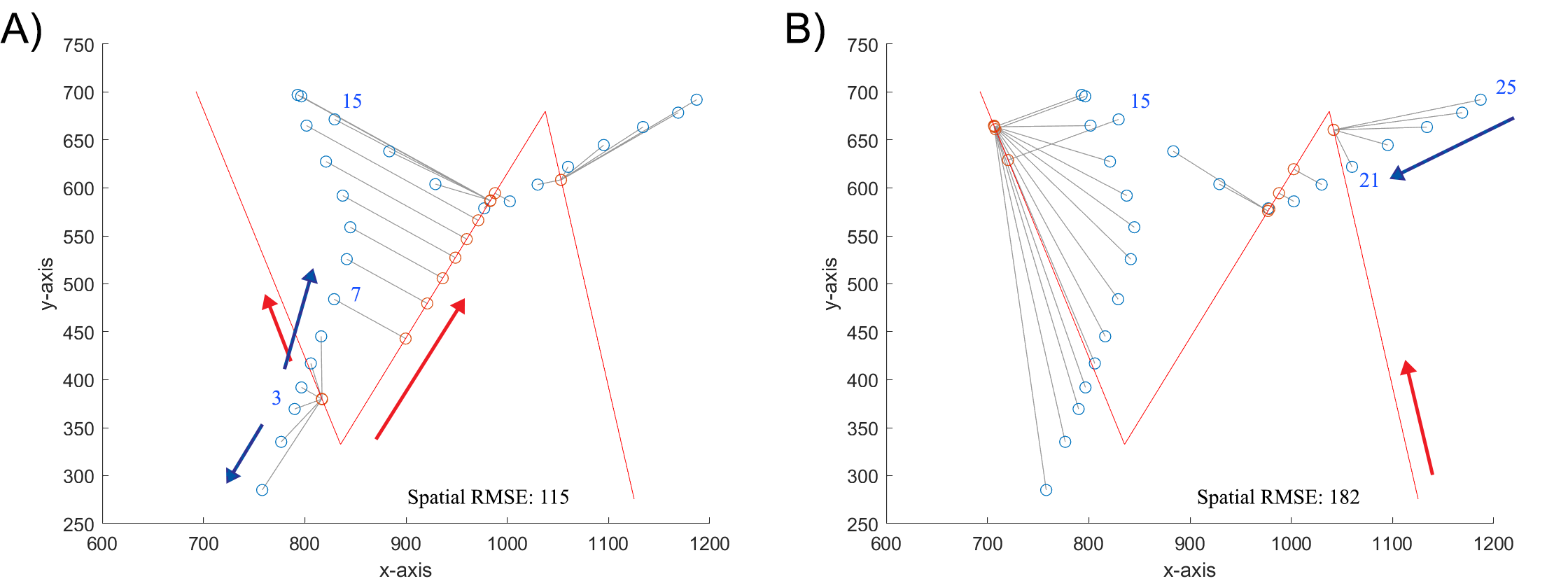
Walkthrough of the spatial matching process

*Note*. Blue dots represent 25 samples of the participant’s response. The red line represents the stimulus path. Red dots represent the stimulus points along this path that each response sample is matched to. Grey lines connect blue and red dots, and depict what part of the stimulus path each blue response sample has matched to. The longer the grey lines, the poorer the spatial match and larger the spatial error. On a subjective assessment, this is a poor response, with both the start and end points going off in the wrong direction. However, the middle of the response does somewhat match the middle segment of the stimulus path, so a best-case spatial match should capture this. To objectively capture this partial match, we do a spatial match starting from each response sample, then choose the best fit.

A) depicts the spatial match that starts at the response sample labelled 3. Response sample 3 is matched to the closest point on the red line, indicated by the leftmost red dot. From there, we work backwards to samples 2 and 1 (i.e., in the direction of the downwards blue arrow). As samples 2 and 1 occur before sample 3, they must match to a stimulus point on the red line that occur before the one that sample 3 matched to in order to preserve temporal continuity, i.e., anywhere along the red line from the leftmost red dot, until the red line ends on the upper left, as indicated by the leftmost red arrow. As everywhere along that section is further away from samples 2 and 1, both have been matched to points that are technically slightly preceding samples 3’s matched point, but for all practical purposes overlap with this point.

With this done, we then move to sample 4 onwards (i.e., in the direction of the upwards blue arrow). Samples 4 to 6 are spatially closest to a section of the stimulus path that occurred before sample 3’s matched point. Such a match would break temporal continuity though, so they are instead also matched to points that are technically after sample 3’s matched point, but practically overlap. Samples 7 to 15 are matched with their spatially closest points along the red line, and form the bulk of the subjectively “correct” response. Samples 16 onwards are matched with similar restrictions to samples 4-6.

B) depicts the attempt to match the same response with the stimulus path, if the matching process started at sample 25 and proceeded in the direction of the blue arrow. This attempt fails to capture the subjective partial match in the middle of the response, and fittingly, results in a much larger spatial RMSE score. Sample 25 is matched to the closest point on the red line, marked by the rightmost red dot. Samples 24 to 21 are closest to segments of the red line before the point at which sample 25 was matched to. However, as this is made impossible by the need to preserve temporal continuity, they are instead matched to the closest point on the line after sample 25’s matching point, i.e., basically right on top of it.

Samples 21 to 16 are straightforward closest matches. However, sample 15 illustrates why this matching attempt fails. Sample 15 is objectively closest to a point near the end of the stimulus path, so matches to that point, and in doing so, restricts all subsequent samples to matching within the small segment on the upper left of the red line, creating very long grey lines.

By following the rules of closest sample-by-sample match and temporal continuity, the best-case fit can be found in a manner that fits our subjective sense of spatial similarity, but remains a purely objective calculation.

**Supplementary Figure 2**


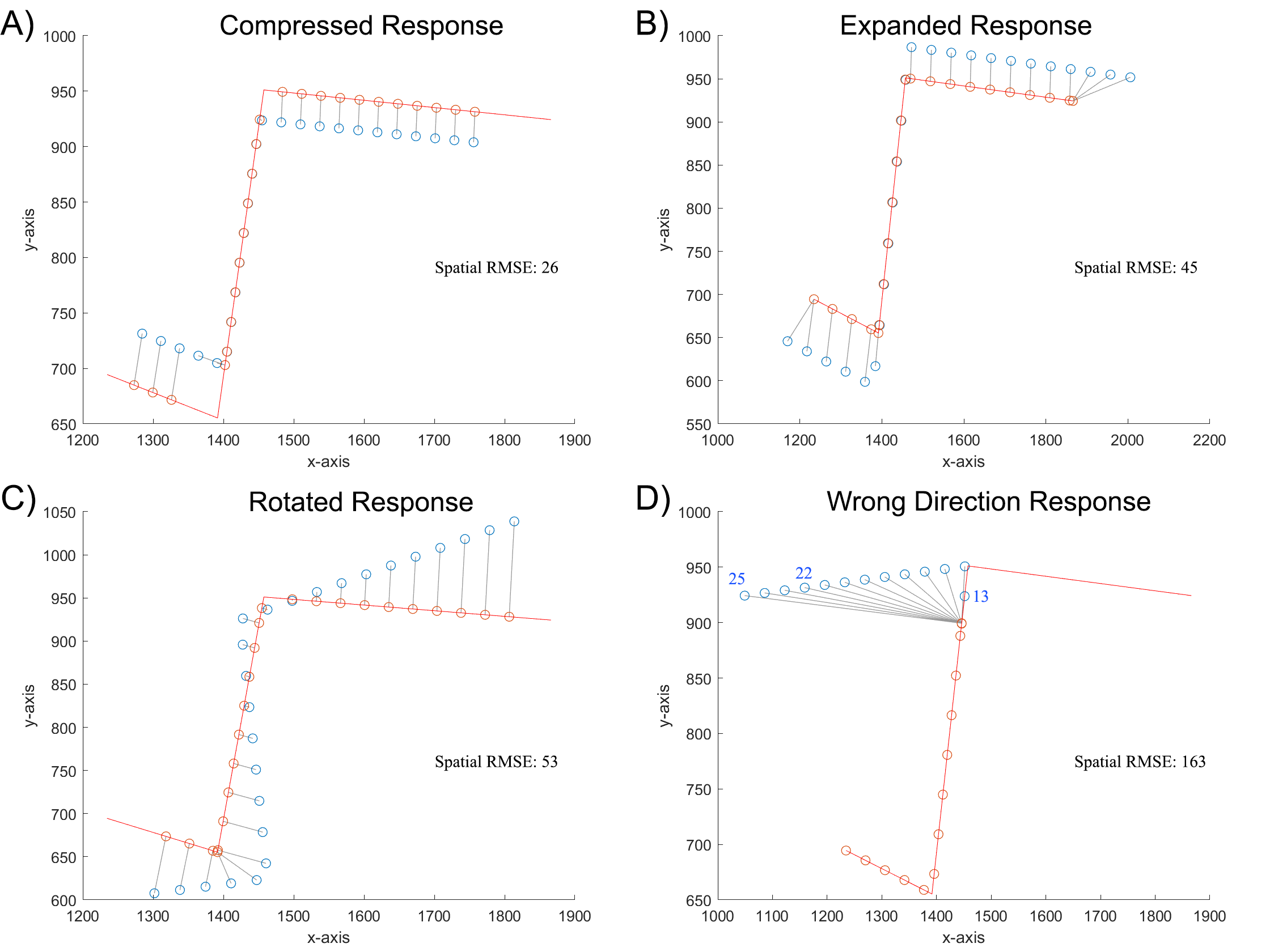
Simulated examples of how the matching process handles spatial distortion.

*Note.* Blue dots represent 25 samples of the participant’s response. The red line represents the stimulus path. Red dots represent the stimulus points along this path that each response sample is matched to. Grey lines connect blue and red dots, and depict what part of the stimulus path each blue response sample has matched to. The longer the grey lines, the poorer the spatial match and larger the spatial error.

A-C depict how the matching process handles simple spatial distortions, illustrated using simulated responses. These simulated responses were created by: (A) compressing the stimulus path to 75% in both the x and y axes, (B) expanding the stimulus path to 125% in both the x and y axes, or (C) rotating the stimulus path 20° anticlockwise about the midpoint. Despite these distortions, each of these remain a subjectively decent response. The objective matching process agrees with this, with spatial RMSE scores that reflect a low degree of spatial error: 26, 45 and 53, respectively (the original stimulus path was taken from Experiment 2, so a histogram of responses can be found in Figure 3C of the main text. For reference, non-lapse responses largely fall between 0 and 100, while lapse responses range from approximately 150 to 300). These low scores are made possible by each sample point being correctly matched to their corresponding segments, with a few exceptions around the turning points. Since the matching process focuses on minimizing the distance for each sample, small but global perturbations only have a minor effect. Also note that the rotated response gives the largest error of the three, because a rotation introduces a larger variation in distances between the matched points and RMSE penalizes infrequent extreme values more than frequent smaller errors.

Large spatial RMSE scores are instead more likely caused by more localized perturbations. D) depicts how the matching process handles a response where a segment has gone off in the opposite direction. The stimulus path was simulated by reflecting the third segment of the stimulus path along the vertical plane. The matching process does its best to match the third segment to the closest point possible, but the resulting grey lines are increasingly long, leading to the large spatial RMSE of 163. Interestingly, the matching process mismatches samples 13 and 14, despite these being basically on the stimulus path. This mismatch enables samples 22 to 25 to match to the last red dot, rather than to the more distant turning point, where it would have had to match to where sample 14 was matched to its closest point. Using a small mismatch to enable the large reduction of another mismatch seems like an intelligent decision, but is simply an emergent property of starting the matching process at sample 22.

Together, these four simulations demonstrate what the range of errors are in spatial RMSE. Smaller errors, i.e., below 100, are often responses that clearly reflect a large degree of memory recall, with some degree of spatial distortion due to general memory deterioration. Large errors, i.e., greater than 100, are more likely due to complete forgetting and subsequent guessing of large segments of the stimulus’ path. A final note that these RMSE values are calculated from pixel differences, so the exact values will depend on the screen resolution (here, 1920 x 1080). A higher screen density will increase the absolute values of the RMSE scores, but the relative values will remain the same.

**Supplementary Figure 3**


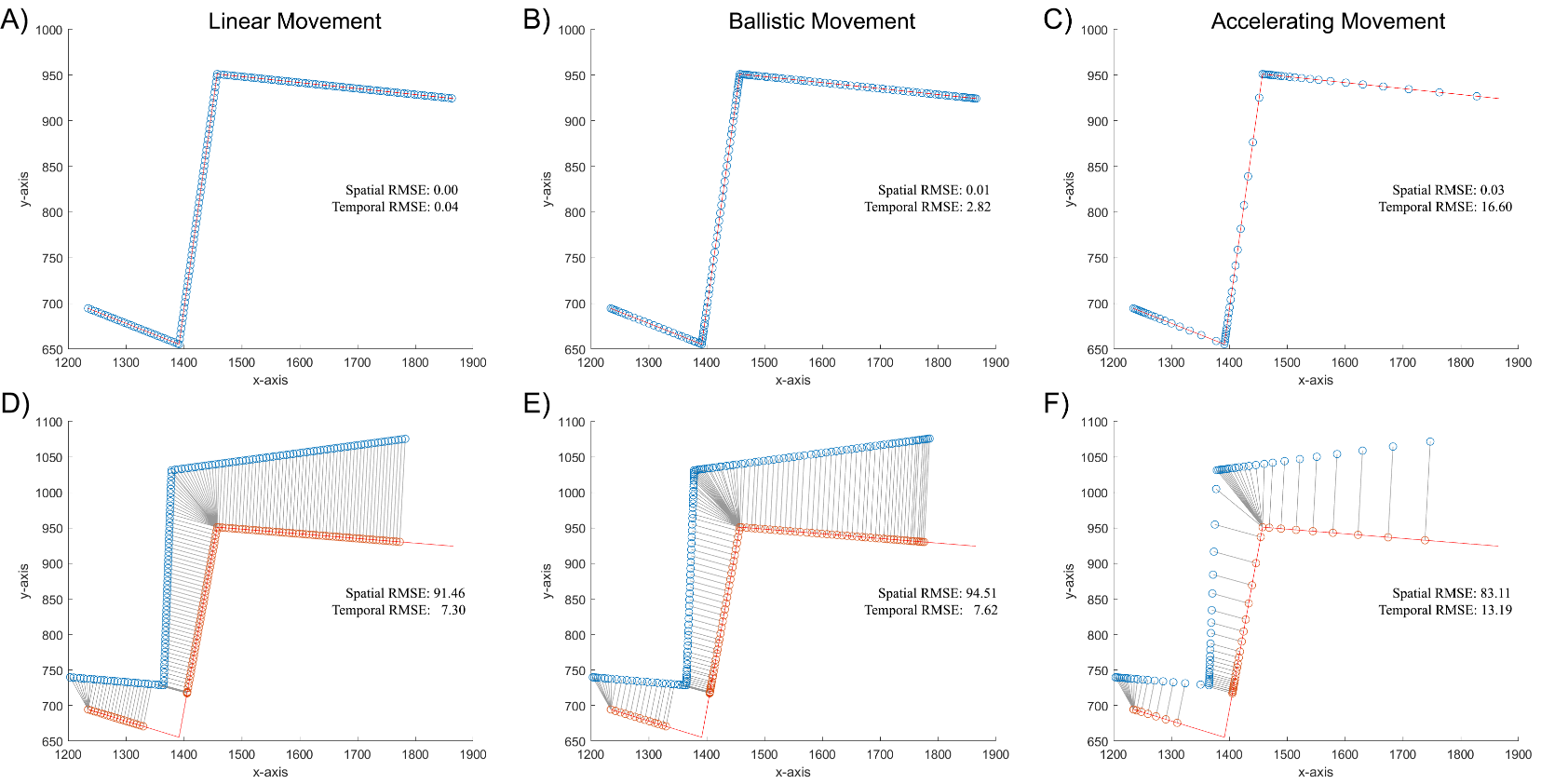
Simulated examples of how the matching process handles temporal distortion.

*Note.* Blue dots represent 150 samples of the participant’s response (150 samples are used here because depicting the accelerating movement requires a large number of samples to visualize). The red line represents the stimulus path. Red dots represent the stimulus points along this path that each response sample is matched to. Grey lines connect blue and red dots, and depict what part of the stimulus path each blue response sample has matched to. The longer the grey lines, the poorer the spatial match and larger the spatial error.

A and D depict a simulation of how the matching process handles a linear movement, where the response advances at a constant pace. In B and E, the response accelerates into the middle of each segment, then decelerates into the end of a segment, as indicated by the blue dots being sparser in the middle and concentrated at the turning points. In C and F, the response accelerates from the start of the segment, before coming to a sudden stop at the end of each segment, as shown by the blue dots getting sparser and sparser as each segment progresses. A-C depict how each of these three movement types affect the matching process if the response is directly on top of the stimulus path, while D-F depict how the matching process handles the three movements in conjunction with a spatial translation (10° anticlockwise rotation from the center of the screen).

From A-C, we can see that a temporal perturbation has no effect on spatial error; it is instead reflected in the temporal RMSE score, which increases from 0 to 3 to 17 as the movement types changes from linear to ballistic to accelerating (the original stimulus path was taken from Experiment 2, so a histogram of responses can be found in Figure 3C of the main text. For reference, non-lapse responses largely fall between 0 and 30 with a positive skew, while lapse responses range will generally sit at 58). From D-F, we can see that that this property changes very little, even with the addition of a spatial perturbation. There is a small variance in the three spatial RMSE scores, from 91 to 95 to 83, due to the differences in sampling. Since the spatial perturbation is a small rotation, the spatial deviation increases as the stimulus proceeds towards the end. Since the accelerating movement bunches up at the start of each segment, and mostly avoids the end of the segment, spatial deviation is smaller than in a linear movement. That said, no amount of fortunate sampling can eliminate the effect of such a spatial perturbation, so the overall error remains.

Note that the effects of natural motor error and movement tendencies largely affect the temporal RMSE scores, with the spatial RMSE scores being a more direct measure of working memory strength. That said, we believe that there is great value to studying the temporal RMSE scores, since effects of natural motor error and movement tendencies should theoretically be greater when working memory strength is low.

**Supplementary Figure 4.**

**
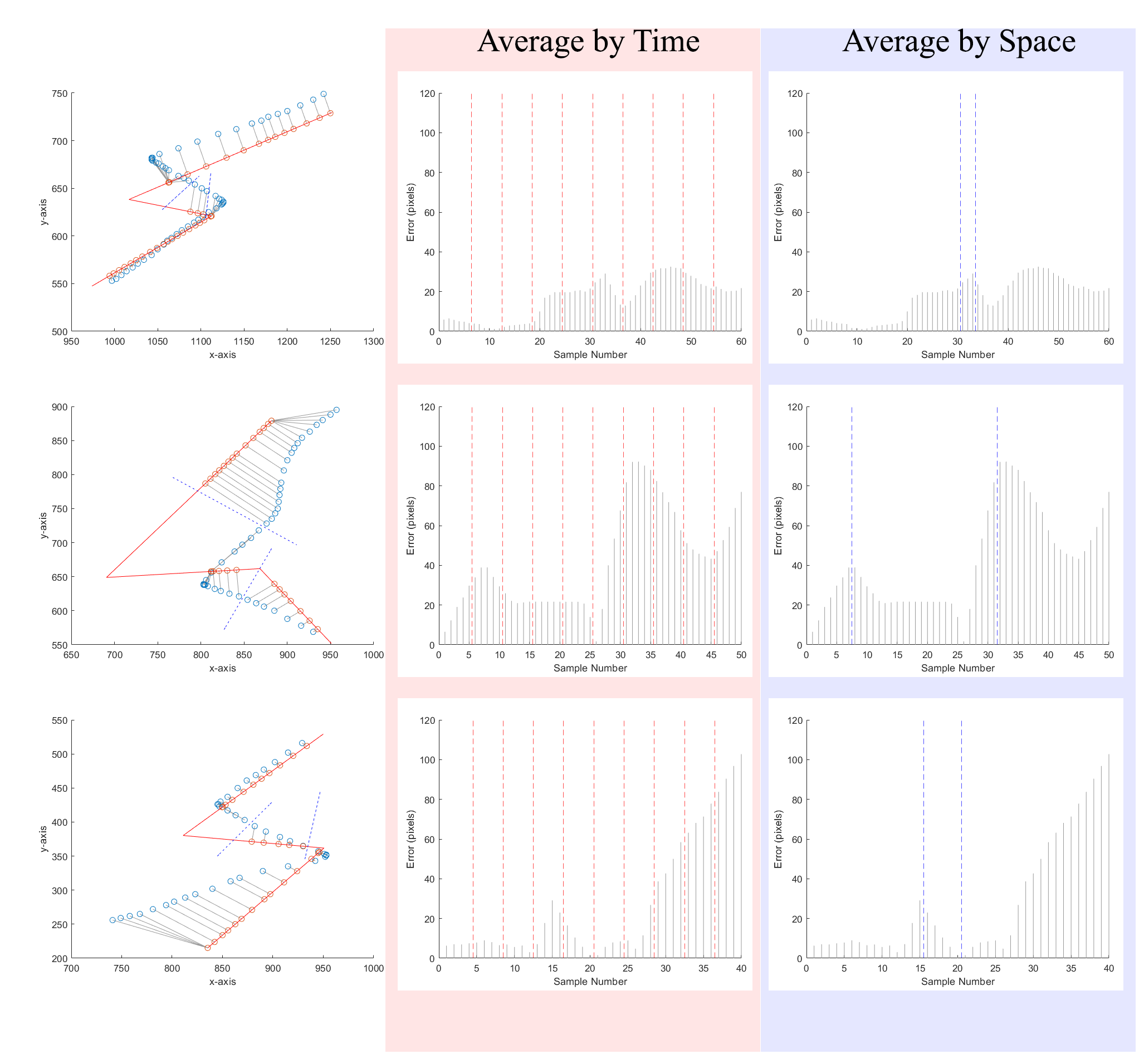
**Illustration of the data averaging process.

*Note*. Each row is an example of a different trial. The first column depicts the response samples (blue dots) to stimulus path (red line) matching process. Each sample of the response is matched to a stimulus point (red dot) along the stimulus path that minimizes the total error on that trial. Grey lines depict the Euclidean distance between the response and its matching stimulus point, with longer lines depicting larger error. The second column depicts how trials are averaged by time. After the matching process, the errors (grey lines) are binned into 100 equally spaced groups (red lines; here only 10 for legibility). The errors in each bin are averaged, then the corresponding bins in all the trials are also averaged to produce Figures 2B and 3D in the main article. The third column depicts how trials are averaged by space. After the matching process, responses are grouped based on which line segment their matching stimulus point lies, as denoted by the dashed blue lines in the first and third columns. Each group is then split using 10 spatially equidistant demarcations, including the start and end points (here there would be a total of 30 demarcations that create 29 bins). Errors between each demarcation are averaged together, then the corresponding bins over all trials are averaged to produce Figures 3C and 3E.

**
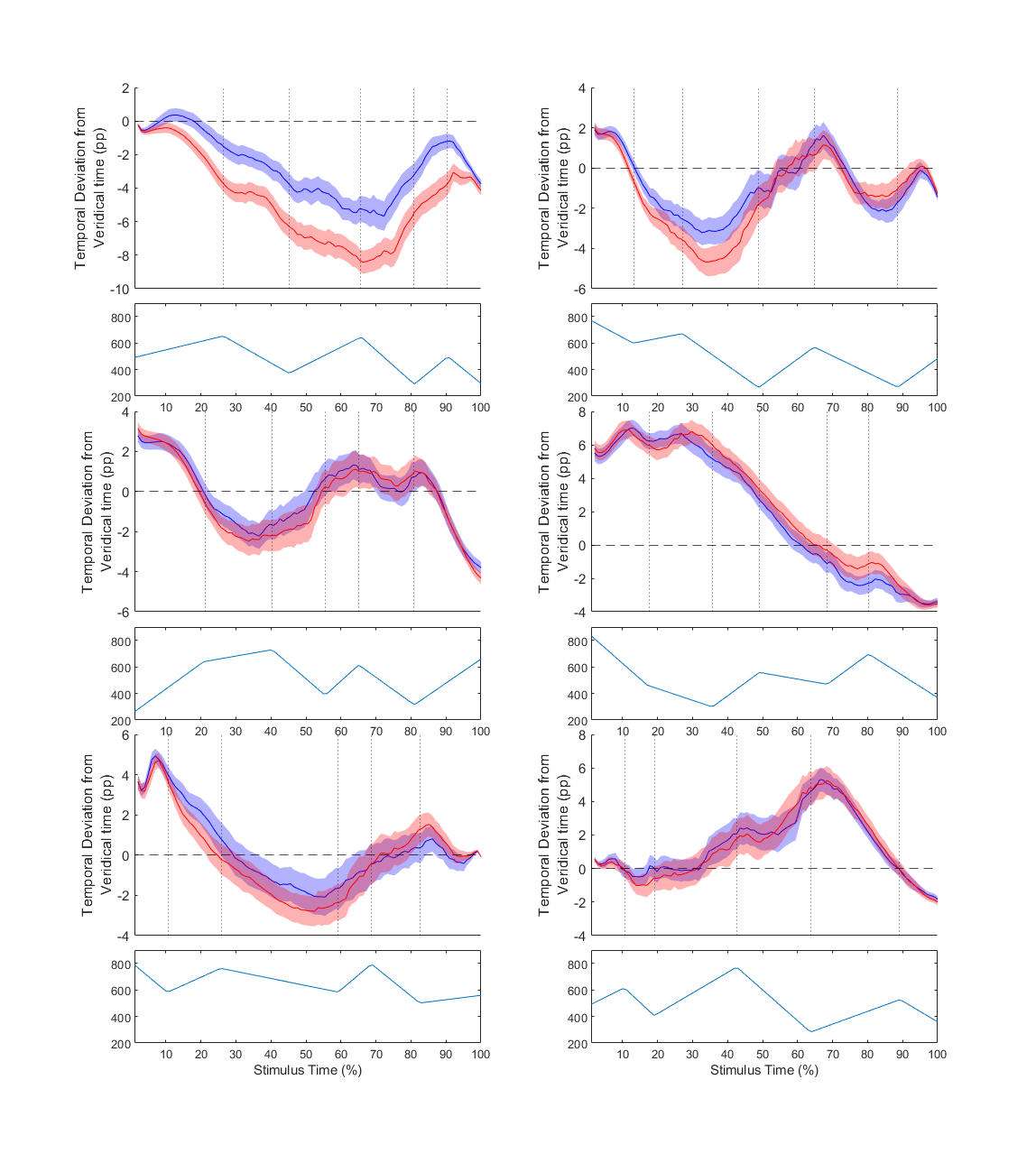
Supplementary Figure 5.**
Temporal deviation from veridical time across the trial for the 6 stimulus paths in Experiment 1.

*Note*. The blue line represents the 1 s retention interval trials, while the red line represents the 10 s retention interval trials. Dotted lines represent turning points in the stimulus path. Underneath each graph is the Y coordinate of the stimulus path (stimulus goes from left to right, so the X coordinate is roughly constant). Error patches represent within-subjects SEM (O’Brien & Cousineau, 2014).
